# Effects of Reflective Processes on Social–Emotional Trait Development in Adulthood: Insights From Two Multi‐Method Studies

**DOI:** 10.1111/jopy.13016

**Published:** 2025-03-06

**Authors:** Gabriela Küchler, Kira S. A. Borgdorf, Corina Aguilar‐Raab, Cornelia Wrzus

**Affiliations:** ^1^ Department of Psychological Aging Research, Psychological Institute Heidelberg University Heidelberg Germany; ^2^ Clinical Psychology, Interaction‐ and Psychotherapy Research, Faculty of Social Sciences University of Mannheim Mannheim Germany; ^3^ Heidelberg University Heidelberg Germany; ^4^ Institute of Medical Psychology University Hospital Heidelberg Heidelberg Germany; ^5^ Network for Aging Research Heidelberg University Heidelberg Germany

**Keywords:** Big Five, comparisons, experiment, IAT, longitudinal personality development, reflective processes

## Abstract

**Objective:**

This research investigates how reflective processes, such as past‐temporal and social comparisons, enhance changes in explicit and implicit self‐concepts of social–emotional traits and whether these effects differ with age.

**Method:**

We conducted two preregistered multi‐method studies to examine whether past‐temporal or social comparisons predict changes in emotional stability and extraversion. In the longitudinal study (*N* = 615, aged 18–84 years), we assessed the frequency of past‐temporal and social comparisons, explicit and implicit self‐concepts of emotional stability and extraversion at two measuring points 6 months apart. In the experimental study (*N* = 231, aged 18–93 years), we elicited trait‐relevant behavioral changes, manipulated past‐temporal versus social comparisons, and assessed explicit and implicit trait self‐concepts before and after the manipulation.

**Results:**

Past‐temporal and social comparisons predicted longitudinal changes in the explicit self‐concept of emotional stability but not extraversion. The experimental study indicated changes in explicit self‐concepts of both traits, regardless of the type of comparison standard. Neither type of comparison predicted changes in implicit self‐concepts. The findings were generalizable across age groups.

**Conclusions:**

This research provides the first evidence that comparisons facilitate the change of explicit but not implicit trait self‐concepts. We discuss short‐term mechanisms of personality development and future directions for examining them experimentally.

## 1 Introduction

In adulthood, personality underlies both stability and dynamic changes that contribute to normative development (Bleidorn et al. 2022) and individual variations in personality trait change (Mund and Neyer 2014; Wagner et al. 2016). Normative personality development largely follows the maturity principle with increases in agreeableness, emotional stability, and conscientiousness across the lifespan (Bleidorn et al. 2022). In addition, extraversion and open‐mindedness decrease, and the pace of personality development is more dynamic in younger than in later adulthood. Importantly, individual differences in the direction and pace of personality development are not yet well understood (Wrzus and Roberts 2017) and may originate from individual differences in the underlying processes. Earlier research focused on explaining these developments primarily through specific life events and transitions, but the findings were mixed, and the effects were smaller than expected (see Bühler et al. 2024, for an overview).

Even though personality changes are well studied, little is known about the processes that cause these changes (Wrzus and Roberts 2017). Everyday experiences could be crucial (Baumert et al. 2017; Quintus et al. 2021; Wrzus and Roberts 2017), but need to be accompanied by associative and reflective processes to gradually leave traces in personality traits (Jackson and Wright 2024; Wrzus and Roberts 2017). Accordingly, the present research focused on reflective processes and their interplay with associative processes in the development of the social–emotional traits of emotional stability and extraversion at different ages.

### 1.1 Processes of Personality Development

Previous literature suggests that everyday experiences gradually shape personality through repeated short‐term processes (Hudson and Roberts 2016; Quintus et al. 2021; Stieger et al. 2021; Wrzus and Roberts 2017). The so‐called TESSERA framework integrates previous work in short‐term processes, which involve recursive sequences of triggering situations, expectancies, states/state expressions, and reactions (TESSERA; Wrzus and Roberts 2017). For example, a trait‐relevant situation (e.g., a social gathering for extraversion) triggers expectations (e.g., acting sociable), elicits trait‐relevant states (e.g., [not] talking to others), and creates reactions in oneself and others (e.g., positive/negative affect). Whereas states corresponding to a person's former trait level contribute to trait stability, trait‐incongruent states contribute to trait changes (Wrzus and Roberts 2017). For example, a rather reserved person acting more extraverted than usual and receiving positive feedback may gradually become more extraverted. Several studies found empirical evidence for these assumptions: Repeated social situations and social behaviors were linked to increases in extraversion (Quintus et al. 2021; Van Zalk et al. 2020), empathic behavior predicted increases in agreeableness (Quintus et al. 2021), and adhering to work ethics predicted increases in conscientiousness (Hudson and Roberts 2016).

Building on the TESSERA framework (Wrzus and Roberts 2017) and the behavioral process model of personality (Back et al. 2009), we propose that personality development manifests at different levels, for example, in explicit and implicit self‐concepts. Explicit self‐concepts represent an individual's propositional evaluation of thoughts, feelings, and behaviors (Back et al. 2009; Gawronski and Bodenhausen 2006; Schmukle et al. 2008), while implicit self‐concepts are represented by automatic associations between situations, thoughts, feelings, and behaviors. Both self‐concepts are associated with each other (Hofmann, Gawronski, et al. 2005) and predict actual behavior in the cases of extraversion and emotional stability (Back et al. 2009). However, these distinct entities develop through different processes over time (Gawronski and Bodenhausen 2006; Gawronski et al. 2017; Quintus et al. 2021; Wrzus et al. 2023): Reflective processes (e.g., comparisons) translate TESSERA sequences mainly into long‐term changes in explicit self‐concepts, and associative processes (e.g., reinforcement learning) primarily into implicit self‐concepts (Wrzus and Roberts 2017).

The present research was conducted as part of a project on social–emotional personality development, focusing on investigating the underlying processes of traits that promote mental health. Thus, we focused on the social–emotional traits of (a) emotional stability, represented by responding less strongly to difficult situations and handling such situations more calmly and positively (Soto and John 2017; Suls and Martin 2005), and (b) extraversion, characterized by sociability, assertiveness, and high levels of energy in social situations (Smillie et al. 2015; Soto and John 2017). Many individuals desire to increase these traits (Hudson et al. 2020; Hudson and Roberts 2014), and of the Big Five traits, emotional stability and extraversion show the strongest positive associations with mental health (Lamers et al. 2012; McNiel and Fleeson 2006; Pletzer et al. 2024) and have been linked to positive life outcomes, such as work success, relationship stability, and increased life expectancy (Roberts et al. 2007).

### 1.2 Change in Explicit Self‐Concepts of Personality Traits: The Role of Reflective Processes

Several theoretical approaches to processes of individual personality development have concluded that self‐reflection is essential for personality change (Baumert et al. 2017; Jackson and Wright 2024; Wrzus and Roberts 2017). More specifically, it is argued that only if trait‐relevant experiences and behaviors are processed through self‐reflection, they can be integrated either by confirming or changing the explicit self‐concept (Baumert et al. 2017; Wrzus and Roberts 2017). Self‐reflection is a process in which individuals examine their thoughts, feelings, behaviors, and experiences (Grant et al. 2002; McAdams 2013). For example, people can ask themselves how their thoughts and behaviors align with their personality, or how they can gradually adapt their behavior based on a desired change (Geukes et al. 2018; Wrzus and Roberts 2017). Moreover, how people evaluate their behavior may contribute to stability or change in their personality. For instance, an anxious person coped better at work after learning new strategies in a workshop. They noticed their improved performance when observing that their colleagues were a lot more stressed about the situation than they were. This could shape their explicit self‐concept if they attribute the change to their own ability to manage stress. However, if they attribute it to external factors like a supportive coworker, their self‐concept probably remains unchanged. As this illustrates, self‐reflection consists mainly of evaluating perceived information, with the individual determining its importance and accuracy (Gawronski and Bodenhausen 2006).

To our knowledge, no studies have explored whether self‐reflection generally promotes trait change across the lifespan. We propose that primarily self‐reflection focused on specific traits and related behaviors influences trait change because frequent comparisons within one domain (e.g., extraversion) do not necessarily imply comparisons in another (e.g., emotional stability; Wrzus et al. 2025). Also, a previous study found that individuals who repeatedly behaved in a conscientious manner and reflected more intensely upon the corresponding situations became more conscientious (Quintus et al. 2021). Interestingly, reflection did not moderate the effects of states on trait change for other Big Five traits. This may be because self‐reflection was reported in general terms, without specifying a focus on particular traits. Thus, the current research aimed at assessing more specific indicators of self‐reflection related to trait‐relevant thoughts, feelings, behaviors, or the trait itself.

In particular, self‐reflections that enhance adjusting the explicit self‐concept should consist of reassessing characteristics by perceiving novel behaviors and evaluating them in comparison to others or another comparison standard (Geukes et al. 2018; Pals 2006; Wrzus and Roberts 2017). Individuals may compare themselves with an ideal self (Higgins 1987), others (i.e., *social comparison* in Festinger, 1954, as cited in Morina 2021), or to themselves in the past (i.e., *past‐temporal comparison* in Albert, 1977, as cited in Morina 2021). We focused on past‐temporal and social comparisons in this research as they seem to be the most frequently used (Morina 2021). For instance, the individual in the prior example might recognize their emotional stability during a hectic day by comparing it to how others handled the situation or how they handled such situations in the past.

To the best of our knowledge, no studies have yet tested how comparisons shape personality self‐concepts across time. But evidence from one‐time experiments shows that singular instances of comparisons do shape the evaluation of several characteristics (e.g., extraversion: Hanko et al. 2010; academic self‐concept: Wolff et al. 2018; task performance: Zell and Alicke 2009). For example, individuals primed to perceive differences evaluated themselves as more extraverted when comparing themselves to a more introverted past self. Conversely, individuals primed to perceive similarities evaluated themselves as more introverted when comparing themselves to a more introverted past self (Hanko et al. 2010). This also highlights the importance of whether individuals compare themselves to a better‐off or worse‐off standard (upward vs. downward comparisons).

In general, upward comparisons are more frequent than downward comparisons on dimensions such as body image, attractiveness, and well‐being (Gerber et al. 2018; McComb et al. 2023; Midgley et al. 2021). In contrast, research showed that individuals generally perceive themselves as better than their average peer (Zell et al. 2020), and as better than themselves in the past regarding their personality traits (Wilson and Ross 2000). Therefore, we assume a preference for downward comparisons in the domain of personality. Importantly, comparisons in other domains have been linked to the desire to improve certain characteristics (Buunk et al. 2007; Gürel et al. 2022) and gain clarity about one's self‐concept (Zaw and Baldwin 2023). Accordingly, regarding personality, comparisons could also be intentionally employed, contrasting current self‐evaluations with those of the past (Hanko et al. 2010) or with other individuals (Wolff et al. 2018).

In sum, we propose that as more specific forms of reflective processes, social and past‐temporal comparisons regarding emotional stability and extraversion may lead to changes in explicit self‐concepts by facilitating the perception of novel thoughts, feelings, and behaviors as a response to experiences, which is the first step in revising one's explicit self‐concept.

### 1.3 Change in the Implicit Self‐Concepts of Personality Traits: The Role of Associative Processes

Implicit self‐concepts evolve and develop through automatic associative processes and are assessed through indirect measures like word categorization tasks (Back et al. 2009). In trait‐relevant situations, various associative processes can come into play. For example, when attending social gatherings, individuals may become more skilled in perceiving social cues through implicit learning (Seger 1994). Also, they may receive positive feedback on their networking skills (feedback learning; Caspi and Roberts 2001) or experience consequences like positive affect and form new friendships (reinforcement learning; Caspi and Roberts 2001). These associative processes may increase the probability of entering similar situations and behaving similarly.

In line with this, repeated reasonable and social behavior predicted increases in the implicit self‐concepts of conscientiousness and extraversion, respectively (Quintus et al. 2021). Most of the research investigating change in implicit self‐concepts has mainly focused on implicit attitudes or self‐esteem rather than personality traits (e.g., Alessandri et al. 2016; Charlesworth and Banaji 2022; Crescentini et al. 2014). Although it is widely tested that explicit and implicit self‐concepts are separate entities that are weakly to moderately linked (Back et al. 2009; Hofmann, Gschwendner, et al. 2005), only a few studies in the field of personality examined how they change simultaneously. While some found that they can change similarly (Egloff et al. 2008; Quintus et al. 2021), others found diverging directions of trait change (Quintus et al. 2021; Wrzus et al. 2023), which could be grounded in the fact that reflective and associative processes do not necessarily work in concert (Gawronski and Bodenhausen 2006). Nevertheless, reflective processes may follow associative activations during experiences (Gawronski and Bodenhausen 2006) and can strengthen the association of explicit and implicit self‐concepts (Egloff et al. 2008; Gschwendner et al. 2006). Accordingly, we tentatively assumed that past‐temporal comparisons may indirectly shape implicit self‐concepts by reinforcing associative activations. For example, a person may automatically react adaptively to a stressor (associatively) and also evaluate this response as more adaptive than previous behaviors in similar situations (reflectively). Strengthening the link between explicit and implicit self‐concepts could help sustain trait changes, as both concepts are different manifestations of the same trait and may influence each other (Hofmann, Gschwendner, et al. 2005). Changing only one self‐concept may lead to a reversal of progress if the other remains unchanged. Additionally, greater congruence between explicit and implicit self‐concepts is desirable, as incongruence has been linked to psychological stress (e.g., Rydell et al. 2008; Schröder‐Abé et al. 2007).

### 1.4 Sources of Age Differences in Personality Change: Reflective and Associative Processes

Strong evidence exists that trait changes are more pronounced in younger compared to older age (Bleidorn et al. 2022), although the underlying reasons remain unclear (Bleidorn and Hopwood 2024; Wrzus et al. 2023). Reflective and associative processes both rely on neuronal flexibility, which declines with age (Bishop et al. 2010; Craik and Bialystok 2006). Consequently, cognitive pursuits become more selective, and learning mechanisms become slower and less effective with age (Head et al. 2008). While establishing a stable identity is crucial in the early stages of life, with older age, people prioritize immediate well‐being and tasks like coping with losses and generativity (e.g., Baltes 1987) over gaining new knowledge for future goals (Socioemotional Selectivity Theory; Carstensen et al. 1999). Accordingly, trait‐specific self‐reflections may be more important and adaptive during younger compared to older age.

Potentially, older individuals may feel less motivated to engage in comparisons due to their greater self‐concept clarity (Diehl and Hay 2011) and weaker desire to change their personality traits (Hudson and Fraley 2016; Quintus et al. 2017). Moreover, this clarity, combined with less desire to change and less cognitive flexibility, may hinder perceiving self‐discrepant information or changing their self‐concept accordingly. Discrepancies between a characteristic's desired and current levels can prompt negative feelings and coping strategies (e.g., Strautman and Higgins 1987). At a younger age, this may not hinder individuals from engaging in comparisons due to their stronger desire for personality change (Hudson and Fraley 2016; Quintus et al. 2017). As individuals age, incongruent information during a comparison may more likely be ignored or disregarded, or they may perceive it as not important or true (Gawronski and Bodenhausen 2006; Pasupathi and Mansour 2006) because they prioritize immediate well‐being (Carstensen et al. 1999).

In line with these assumptions, previous research demonstrated that older individuals engage in fewer comparisons regarding their abilities, opinions, and personality (Callan et al. 2015; Küchler et al. 2025) than younger individuals, but in more comparisons related to health (Mehlsen et al. 2019). These findings suggest that the focus of self‐reflections may shift toward characteristics one desires or needs to improve at that point in life (Küchler et al. 2025).

Also, prior research found evidence that could suggest that the effects of comparisons on an individual's self‐concept could vary with age. For example, older individuals compared to younger individuals prioritized consistency over change in self‐reflection (McLean 2008; Pasupathi et al. 2006; Sneed and Whitbourne 2003). Also, younger individuals were more prone to identity accommodation (i.e., making changes in the self‐concept), whereas older individuals were more prone to identity assimilation (i.e., maintaining self‐consistency; Sneed and Whitbourne 2003).

To our knowledge, no study has investigated age differences in the effect of trait‐specific comparisons on trait change. We suggest that, generally, comparisons should have stronger effects on individuals with younger as opposed to older ages, consequentially leading to greater change in their explicit self‐concepts. Moreover, although we generally expect a lower frequency of comparisons in older individuals, with age, people may particularly engage in fewer social comparisons because of less diverse social interactions (Weber et al. 2020). Furthermore, with age, the better‐than‐average effect typically associated with social comparisons regarding personality diminishes (Zell et al. 2020), indicating that older individuals may not perceive themselves as superior to others, thereby potentially avoiding social comparisons even more than past‐temporal comparisons to evade psychological conflict. Nevertheless, in contrast to themselves in the past, people tend to perceive their current selves as superior regarding traits such as social skills, open‐mindedness, and reliability (Wilson and Ross 2001). Accordingly, past‐temporal comparisons likely still exert a stronger influence on older individuals than social comparisons do.

Regarding implicit self‐concepts, a similar pattern is expected, considering the impact of aging on associative processes. Some previous research found inconsistent age differences in the link between trait‐relevant states, reactions, and personality change (Quintus et al. 2021). However, a substantial body of literature has shown that, compared with younger individuals, older individuals exhibit disruptions in associative learning (Mutter et al. 2019), reinforcement learning (Cutler et al. 2021), and general learning from experiences (Mata et al. 2011). Accordingly, associative processes linked to personality development may be less effective, resulting in less change in implicit self‐concepts with older age.

### 1.5 The Present Research

In the present research, we employed a multi‐method approach to investigate the effects of comparisons as reflective processes on changes in emotional stability and extraversion at different ages. We aimed to answer three main research questions: First, given the lack of evidence for the effects of comparisons on personality development, we investigated whether comparisons enhance changes in emotional stability and extraversion and simultaneously tested potential differences in the effects of past‐temporal and social comparisons. Second, we explored changes in explicit and implicit self‐concepts, assuming that social comparisons should affect explicit but not implicit self‐concepts. Third, to add to the literature on sources of age differences in personality development, we explored whether comparisons enhance trait changes less strongly in individuals with older compared to younger ages. To address these research questions, we implemented two studies with age‐heterogeneous samples and two distinct designs. In Study 1, we aimed to investigate the long‐term effects of comparisons on personality development by using a longitudinal design. Because short‐term processes elicited in specific situations could not be explored in this design, we further employed an experimental design in Study 2.

In Study 1, we employed a longitudinal design over 6 months in two countries (United States and Germany) to move beyond single‐country studies. The frequency of past‐temporal and social comparisons is trait‐specific and has a similar retest stability as traits (Wrzus et al. 2025). Importantly, we expected individuals to have sufficient occasions for comparisons because fluctuations of trait‐relevant states are manifested in everyday behavior (Quintus et al. 2021; Wrzus and Roberts 2017), and most people wish to improve their emotional stability and extraversion (Hudson and Roberts 2014). Accordingly, we hypothesized (H1a) that with a more pronounced initial frequency of past‐temporal comparisons, the explicit and implicit self‐concepts of emotional stability and extraversion change more strongly over time. Moreover, (H1b) with a more pronounced initial frequency of social comparisons, the explicit (but not implicit) self‐concepts of these traits change more strongly over time. We assumed that social comparisons could be particularly relevant for extraversion because it is an interpersonal trait, particularly visible in social interactions (Costa and McCrae 1989). Accordingly, we hypothesized (H2) that changes in extraversion would be more pronounced with a more pronounced initial frequency of social comparisons relative to past‐temporal comparisons. We expected no differences between both comparison standards for changes in emotional stability. Last, we hypothesized (H3) that changes in the explicit and implicit self‐concepts of emotional stability and extraversion are more pronounced among younger individuals compared to older individuals.

We did not have any hypotheses for cross‐country differences related to the research questions investigated. However, in exploratory analyses, we tested whether the effects of comparisons and age on trait change were moderated by the country of residence. Also, we conducted exploratory analyses with general self‐reflections as control variables to determine whether our assumption that exclusively trait‐specific comparisons, rather than general self‐reflection tendencies, predicted changes in explicit trait self‐concepts.

In Study 2, we aimed to induce short‐term changes in personality self‐concepts. As explained in more detail in the Procedure section of Study 2, we created triggering situations and induced states relevant to emotional stability and extraversion. Then, we randomly assigned participants to compare themselves either to others or to themselves in the past. Importantly, the experiment included a social interaction during stress induction and reduction. Because previous research showed that extraversion is more influenced by behavioral changes than emotional stability (Quintus et al. 2021), we formulated the hypothesis (H1a) that changes in extraversion are more pronounced compared to changes in emotional stability. Moreover, we again hypothesized (H1b) that changes in extraversion are more pronounced after social comparisons relative to past‐temporal comparisons. In line with H2 of Study 1, no difference was expected for emotional stability. In the experiment, the TESSERA framework sequence was enacted once, and because the establishment of associative memory usually needs more repetitions (for an overview, see Gawronski and Bodenhausen 2006), we hypothesized (H2a) that explicit trait self‐concepts change more compared to implicit trait self‐concepts. Furthermore, we expected (H2b) that changes in implicit self‐concepts are not affected by the comparison standard. Again, we hypothesized (H3a) that personality changes are more pronounced among younger adults compared to older adults. Furthermore, we expected (H3b) that personality changes among younger adults are more pronounced when based on social comparisons compared to past‐temporal comparisons, whereas personality changes among older adults are more pronounced based on past‐temporal comparisons compared to social comparisons.

## 2 Study 1: Effects of Past‐Temporal and Social Comparisons on Longitudinal Trait Change

### 2.1 Method

The data of this manuscript were collected within a larger project. The study design, sample rationale, hypotheses, and data analyses were preregistered after the data collection of T1 but prior to T2, and none of the hypotheses can be tested without data from T2: https://osf.io/mrvps. Deviations, all minor, from the preregistration are explained in Table S1. The wording of the hypotheses was exclusively changed for grammatical correctness and consistency. The data, code, and materials are available at https://osf.io/rwkjf/. The Ethics Committee of the Psychological Institute of Heidelberg University approved the research project (Wrzus 2019 1/1) and all participants gave informed consent before participation.

#### 2.1.1 Procedure

To recruit an age‐ and gender‐heterogenous sample with diverse socioeconomic backgrounds, individuals from the United States and Germany were invited to participate in the online study (SoSci Survey; Leiner 2021) on “behavior and experiences in everyday life” via the crowdsourcing platform Clickworker. The participants were informed that the study consisted of three waves of data collection, which would be compensated with a total of €14.50/ $16.80, respectively. In Germany, the data were sampled in August 2021 (T1), September 2021 (Retest), and February 2022 (T2), and in the United States in October 2021 (T1), November (Retest), and April 2022 (T2). Only T1 and T2 contained all relevant variables for this manuscript. The time lag of 6 months was chosen because previous studies (e.g., Quintus et al. 2021) showed that it is sufficient to observe the effects of short‐term processes of personality development in correlational studies (without interventions). After giving informed consent, the participants provided demographic information, completed questionnaires and implicit association tests, and then generated a personalized code for data matching.

#### 2.1.2 Participants

We aimed for 330 participants per country, expecting an attrition rate of 10%. The power analysis was based on a different research question of this project, which involved a more complex model with interaction effects. For that analysis, we aimed for *N* = 300 with a power of 1−*β* = 0.95 and an alpha level of *α* = 0.05, assuming a main effect of 0.20 and an interaction effect of 0.10. To participate in the study, individuals had to be at least 18 years old, have PC or laptop access, and have good English or German language skills, respectively. We employed quotas for five age groups (18–30, 31–44, 45–58, 59–72, and 73–86 years), separately for individuals identifying as male or female, to gain a gender‐ and age‐heterogeneous sample.

A total of 648 individuals participated at T1 (321 in the United States and 327 in Germany). To preserve data quality, we excluded responses from participants that had an unrealistically low survey duration (below 50% of the estimated time, i.e., below 15 min) or a combination of various suspicious response behaviors (e.g., giving the same answer more than 10 times in a row; Yentes and Wilhelm 2018) or more than two failed attention check items (as described in Meade and Craig 2012; see preregistration). After the exclusion of 33 participants (19 US and 14 German participants), the final sample of *N* = 615 included 313 German participants aged 18–84 years (*M*_age_ = 43.32, SD_age_ = 14.91) and 304 US participants aged 18–78 years (*M*_age_ = 41.32, SD_age_ = 13.94; for more demographic information, see Table S2). At T2, 320 adults participated, and after applying the same exclusion criteria as T1, the final sample of 309 participants included 228 German participants (attrition rate: 26.84%) aged 18–84 years (*M*_age_ = 46.09, SD_age_ = 14.57) and 81 US participants (attrition rate: 73.18%) aged 19–68 years (*M*_age_ = 41.98, SD_age_ = 11.57).

Attrition analyses showed that individuals who did not participate in T2 differed significantly from the remaining participants on some variables. Opposed to non‐completers, completers were older, more emotionally stable, and compared themselves less with others regarding extraversion and less with themselves regarding emotional stability. There were no differences regarding other variables (see Table S3 for more details).

#### 2.1.3 Measures

##### 2.1.3.1 Explicit Self‐Concepts

We measured explicit self‐concepts with the Big Five Inventory‐2 (BFI‐2; Danner et al. 2019; Soto and John 2017). Twelve items each measured emotional stability^1^ (e.g., “I am someone who is relaxed, handles stress well”; ω _T1_ = 0.91, range ω _T1_: 0.89–0.92; ω _T2_ = 0.91, range ω_T2_: 0.90–0.93) and extraversion (e.g., “I am someone who is talkative”; ω_T1_ = 0.86, range ω_T1_: 0.83–0.87; ω_T2_ = 0.87, range ω_T2_: 0.84–0.89). Participants responded on a 5‐point Likert scale from 1 (*disagree strongly*) to 5 (*agree strongly*).

##### 2.1.3.2 Implicit Self‐Concepts

We used an implicit association test (IAT) to assess implicit self‐concepts of emotional stability and extraversion (Schmukle et al. 2008), which was validated in previous research (e.g., Back et al. 2009; Greenwald et al. 2009). The IAT consists of computer‐based word‐sorting tasks with three practice blocks of 20 trials (Blocks 1, 2, and 4) and two test blocks (Blocks 3 and 5) with 60 trials for each trait (Greenwald et al. 2003; Richetin et al. 2015). Target categories labeled “me” and “others” consisted of five stimuli each (e.g., I, myself, their, your), while attribute categories (traits) had five stimuli each, differing for anxiety versus calmness (e.g., calm, nervous) for emotional stability and extraversion versus introversion (e.g., talkative, reserved) for extraversion. Target and attribute stimuli were interchanged in the test blocks 3 and 5, the order of words was randomized across blocks, and stimuli in a given block were repeated (without replacement) until the specified number of trials was reached. The differences in reaction times when sorting words related to high trait levels with “me” (vs. “others”) or low trait levels with “me” (vs. “others”) were considered indicative of implicit associations with specific trait levels (e.g., Schmukle et al. 2008). Values representing implicit self‐concepts (D_2_ scores) were calculated with built‐in error penalties and winsorized reaction times < 300 ms and > 10,000 ms (Greenwald et al. 2003; Richetin et al. 2015). For latent change analysis, even and odd stimuli of all trials were split into two parcels (e.g., Quintus et al. 2021; Schmukle et al. 2008) and neuroticism scores were reversed to represent emotional stability. Split‐half reliabilities were acceptable for both traits, with internal consistencies of 0.81 (T1) and 0.73 (T2) for emotional stability, and 0.89 (T1) and 0.88 (T2) for extraversion.

##### 2.1.3.3 Past‐Temporal and Social Comparisons

Based on previous research, we developed a questionnaire to assess the frequency of past‐temporal and social comparisons of emotional stability and extraversion (Wayment and Taylor 1995; Wilson and Ross 2000; Wrzus et al. 2025). Items were translated into English for the US sample, and a professional translator checked the items for spelling, grammar, and cultural appropriateness.

At T1, two items per personality trait measured comparison frequency (four items in total): One item assessed the frequency of past‐temporal comparisons (“How often do you compare yourself with yourself in the past regarding how calm/sociable you are?”) and social comparisons (“How often do you compare yourself with other people regarding how calm/sociable you are?”). Participants responded on a 7‐point Likert scale from 1 (*never*) to 7 (*always*).

##### 2.1.3.4 Control Variables

Ruminative self‐reflections (e.g., “I often reflect on episodes in my life that I should no longer concern myself with”; ω = 0.93, range ω: 0.92–0.94) and explorative self‐reflections (e.g., “I love analyzing why I do things”; ω = 0.88, range ω: 0.87–0.91) were assessed using the Reflection‐Rumination Questionnaire (Post 2004; Trapnell and Campbell 1999), which includes 12 items for each scale. Participants provided their responses on a 5‐point Likert scale ranging from 1 (*strongly disagree*) to 5 (*strongly agree*).

#### 2.1.4 Analytic Strategy

**FIGURE 1:**
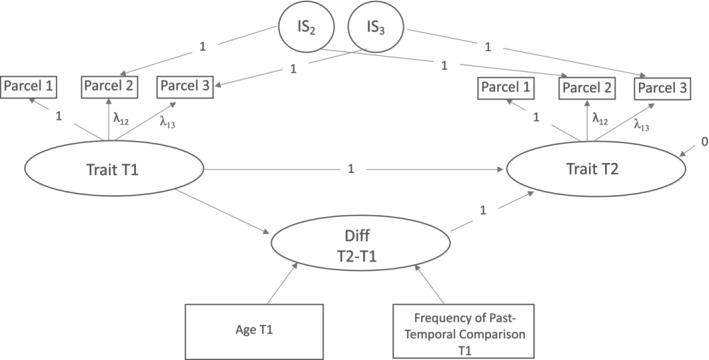
Latent change model of explicit self‐concepts, as an example for testing H1a. Latent traits were estimated with three indicators (parcels) for each measurement point (T1 and T2). Measurement invariance was established by constraining intercepts (not displayed) and factor loadings to be equal for each measurement. Repeated method effects were accounted for by indicator‐specific method factors (IS2, IS3). The latent variable Diff T2–T1 reflects the amount of latent change in traits from T1 to T2. Latent trait change was predicted by age, and past‐temporal comparison frequency indicated at T1.

There were no outliers (*M* ± 3 SD). All predictors were z‐standardized. The country of residence was entered as a control variable in all analyses (−1 = United States, 1 = Germany). We used RStudio Version 1.4.1106 for data preparation and control analyses (RStudio Team 2021; see Table S4 for used packages). We tested measurement invariance for each trait and type of self‐concept, and strong measurement invariance held in each measurement model (Chen 2007; see Table S5 for details). To test our hypotheses, we applied latent change analysis (Geiser 2011; e.g., see Figure 1) in Mplus Version 8.6 (Muthén and Muthén 1998–2017). Personality traits at T1 and T2 were modeled as latent variables. Latent explicit self‐concepts were modeled with three content‐based parcels (means of four items each), representing the three facets of emotional stability (anxiety, depression, emotional volatility, reversed) and extraversion (sociability, assertiveness, and energy level), respectively (Matsunaga 2008; Soto and John 2017). The latent implicit self‐concepts were modeled with two parcels for T1 and T2, in line with split‐half D_2_ scores (Schmukle et al. 2008). The latent change score was modeled by establishing the latent trait score at T2, which was perfectly regressed on the latent trait score at T1 and the latent change score (Geiser 2011). We computed separate models for each trait for the explicit and implicit self‐concepts with the following predictors: past‐temporal comparison and age (Model 1), social comparison and age (Model 2), and only age (Model 3) as manifest predictors of latent trait change. In all analyses, we used the maximum likelihood estimator with robust standard errors that are robust against normality violations (Muthén and Muthén 1998–2017). We accounted for longitudinal method effects by indicator‐specific uncorrelated method factors. All models were defined with invariant factor loadings and intercepts (i.e., strong factorial invariance). According to best practices, we report one‐tailed *p* values for directional hypotheses and two‐tailed *p* values for non‐directional hypotheses (Cho and Abe 2013; Lakens 2022).

### 2.2 Results Study 1: Comparisons as Predictors of Longitudinal Trait Changes

Table 1 displays descriptive information and correlations of the explicit and implicit self‐concepts of emotional stability and extraversion at T1 and T2, as well as past‐temporal and social comparisons of each trait, age, and country at T1. The results of the latent change analyses are displayed in Table 2. Model fit indices of all models are displayed in Table 3.

**TABLE 1:** Descriptive information and correlations between variables of interest.

	1	2	3	4	5	6	7	8	9	10	11	12	13
1. ES explicit T1													
2. ES explicit T2	**0.90**												
3. ES implicit T1	0.18	0.21											
4. ES implicit T2	0.19	0.20	**0.43**										
5. EX explicit T1	0.39	0.37	0.06	0.07									
6. EX explicit T2	0.35	0.36	0.04	0.04	**0.89**								
7. EX implicit T1	0.21	0.17	−0.11	−0.04	0.40	0.39							
8. EX implicit T2	0.13	0.10	−0.01	−0.01	0.27	0.32	**0.49**						
9. Past‐temporal comparison ES	−0.23	−0.17	0.05	0	0.03	0.04	0.05	0.02					
10. Social comparison ES	−0.29	−0.23	0.00	−0.03	−0.01	0.00	0.08	0.08	0.69				
11. Past‐temporal comparison EX	−0.31	−0.27	−0.05	−0.01	−0.08	−0.07	−0.06	−0.05	0.57	0.45			
12. Social comparison EX	−0.34	−0.33	−0.13	−0.11	−0.26	−0.25	−0.06	−0.07	0.40	0.49	0.63		
13. Age	0.24	0.21	0.14	0.20	0.11	0.11	0.01	−0.02	−0.13	−0.18	−0.16	−0.18	
14. Country	0.12	0.11	0.06	0	0.13	0.15	0.10	0.07	−0.04	−0.08	−0.14	−0.15	0.13
*M* (SD)	3.20 (0.79)	3.18 (0.77)	0.34 (0.36)	0.33 (0.33)	2.96 (0.69)	2.95 (0.68)	−0.23 (0.51)	−0.24 (0.44)	3.25 (1.50)	3.10 (1.55)	3.45 (1.57)	3.52 (1.52)	45.01 (14.03)

*Note:* Country: 1 = Germany, −1 = United States. All correlation coefficients larger than |0.15| are significant with *p* < 0.05. Rank‐order correlations are bolded.

Abbreviations: ES, emotional stability; EX, extraversion.

**TABLE 2:** Past‐temporal and social comparisons, and age predicting longitudinal change in the explicit and implicit self‐concepts of emotional stability and extraversion.

Model		Emotional stability		Extraversion	
		Estimate [95% CI]	*p*	Estimate [95% CI]	*p*
**Explicit**					
M1	Change T2–T1	−0.008^a^ [−0.063, 0.046]	0.761	−0.037^a^ [−0.103, 0.030]	0.280
	PTCOMP	0.055 [0.016, ∞]	**0.010**	0.012 [−0.028, ∞]	0.316
	Age	−0.033 [−∞, 0.007]	0.090	−0.006 [−∞, 0.035]	0.400
M2	Change T2–T1	−0.009^a^ [−0.064, 0.045]	0.735	−0.036^a^ [−0.102, 0.030]	0.289
	SCOMP	0.048 [0.009, ∞]	**0.023**	−0.002 [−0.043, ∞]	0.471
	Age	−0.032 [−∞, 0.009]	0.100	−0.008 [−∞, 0.034]	0.371
M3	Change T2–T1	−0.008^a^ [−0.062, 0.047]	0.788	−0.036^a^ [−0.103, 0.031]	0.293
	Age	−0.040 [−∞, 0.000]	**0.048**	−0.008 [−∞, 0.031]	0.367
**Implicit**					
M1	Change T2–T1	0.001^a^ [−0.050, 0.053]	0.967	0.001^a^ [−0.059, 0.060]	0.982
	PTCOMP	−0.002^a^ [−0.038, 0.034]	0.914	−0.005^a^ [−0.051, 0.041]	0.838
	Age	0.019 [−∞, 0.054]	0.187	−0.019 [−∞, 0.030]	0.262
M2	Change T2–T1	0.001^a^ [−0.050, 0.053]	0.963	0.001^a^ [−0.059, 0.061]	0.984
	SCOMP	−0.005^a^ [−0.043, 0.033]	0.787	−0.004^a^ [−0.051, 0.043]	0.870
	Age	0.018 [−∞, 0.053]	0.194	−0.019 [−∞, 0.030]	0.262
M3	Change T2–T1	0.001^a^ [−0.050, 0.052]	0.967	0.000^a^ [−0.060, 0.060]	0.988
	Age	0.019 [−∞, 0.054]	0.182	−0.018 [−∞, 0.030]	0.268

*Note: N* = 309. M1 = Model 1, M2 = Model 2, M3 = Model 3. Significant *p* values (*p* < 0.05) are bolded.

Abbreviations: PTCOMP, past‐temporal comparison; SCOMP, social comparison.

^a^ Undirected hypothesis.

**TABLE 3:** Model fit indices of models predicting longitudinal change in the explicit and implicit self‐concepts of emotional stability and extraversion.

Model	*χ* ^2^	CFI	TLI	RMSEA	SRMR
**ES explicit**					
M1 (PTCOMP + Age)	25.782	0.993	0.986	0.044	0.024
M2 (SCOMP + Age)	27.110	0.992	0.984	0.047	0.025
M3 (Age)	24.992	0.992	0.985	0.050	0.026
**EX explicit**					
M1 (PTCOMP + Age)	26.869	0.990	0.979	0.047	0.034
M2 (SCOMP + Age)	25.453	0.991	0.982	0.044	0.035
M3 (Age)	25.033	0.989	0.980	0.051	0.038
**ES implicit**					
M1 (PTCOMP + Age)	12.998	0.988	0.976	0.038	0.031
M2 (SCOMP + Age)	21.061	0.964	0.929	0.066	0.042
M3 (Age)	4.291	0.999	0.997	0.015	0.018
**EX implicit**					
M1 (PTCOMP + Age)	18.857	0.985	0.970	0.060	0.041
M2 (SCOMP + Age)	24.515	0.976	0.953	0.075	0.048
M3 (Age)	2.517	1.000	1.000	0.000	0.007

Abbreviations: CFI, comparative fit index; ES, emotional stability; EX, extraversion; M1, Model 1; M2, Model 2; M3, Model 3; PTCOMP, past‐temporal comparison; RMSEA, root mean square error of approximation; SCOMP, social comparison; SRMR, standardized root mean square residual; TLI, Tucker–Lewis index.

On average, no significant mean‐level trait change occurred in either trait or type of self‐concept. Regarding individual differences, we found partial support for H1a: Individuals who were more prone to compare themselves with their past selves at T1 increased their explicit self‐concept of emotional stability more strongly. However, this was not the case for the explicit self‐concept of extraversion or the implicit self‐concepts of both traits. As predicted in H1b, individuals who were more prone to compare themselves with others increased their explicit emotional stability more strongly. Contrary to our predictions, this was not the case for extraversion. Moreover, as expected, social comparisons did not predict change in the implicit self‐concept of both traits.

In line with H2, both types of comparisons affected changes in emotional stability to a similar extent. Contrary to our predictions, social comparisons did not affect changes in extraversion more strongly than past‐temporal comparisons because neither type of comparison was associated with trait changes. Contrary to H3, changes in explicit and implicit self‐concepts were not more pronounced among individuals with younger ages compared to individuals with older ages.

Exploratory analyses revealed no significant differences between the United States and Germany in the associations between trait‐specific comparisons and the corresponding explicit and implicit self‐concepts (see Table S6 for details). Age‐related differences in the explicit self‐concepts of emotional stability and extraversion, as well as the implicit self‐concept of emotional stability, were also similar in both countries. For the implicit self‐concept of extraversion, there was a significant negative main effect of age and a significant positive interaction effect between age and country. The interaction effect indicated that changes in the implicit self‐concept of extraversion were less pronounced with older age in the United States (coded as −1), and this effect was weaker in Germany (coded as 1). Exploratory analyses with general self‐reflection tendencies as control variables showed that the effects of the original analyses remained unchanged, and general self‐reflections did not predict trait change (see Table S7 for detailed results).

## 3 Study 2: Effects of an Experimental Manipulation of Comparison Standards on Short‐Term Trait Change

### 3.1 Method

This study consisted of a total of three measurement points. As preregistered, only the first (T1) and second (T2) measurements were analyzed for the current research questions. Data were collected from March 2022 to June 2023 in Heidelberg, Germany. The preregistration of the study design, sample rationale, hypotheses, and analyses prior to data collection are available at https://osf.io/cy76w. The codebook, data sets, scripts, and experimental manipulations are available at https://osf.io/rwkjf/. Minor linguistic deviations from the preregistration can be found in Table S8. The study adhered to the principles of the Declaration of Helsinki, was approved by the Ethics Committee of the Psychological Institute of Heidelberg University, and all participants gave informed consent before participation.

#### 3.1.1 Participants

Participants were recruited for the study “Heart and Mind,” allegedly on age and personality differences in cognitive tasks as well as the role of mood in cardiac activity. They were informed that there were three sessions, two online and one in the laboratory, with a full debriefing after the final session. Furthermore, they were informed that the laboratory session included cognitive and social interaction tasks, with a cardiac activity measurement. Recruitment methods included public advertisement via flyers and posters, social media, newspaper articles, and presentations in educational institutions and senior citizen centers. Participants were compensated with €20.

Power analyses were based on between‐effects of the past‐temporal versus social comparison condition on personality trait change. To detect effects of medium size (0.20) with 1−*β* = 0.85, *α* = 0.05, we needed to recruit a minimum of 230 participants (*n* = 115 younger adults, *n* = 115 older adults). We sampled participants aged 18–33 years (younger adults) and 60+ years (older adults) to maximize the power for detecting age differences.^2^ The selected age range aligns with general conventions defining younger and older adulthood (see Freund and Ritter 2009).

We established five inclusion criteria: an age of 18–33 or 60+ years, PC or laptop access, good German language skills, no background in psychology (e.g., students, psychologists, or therapists), and no cardiac arrhythmia or implanted pacemaker. After data collection, we applied the same preregistered exclusion criteria as in Study 1. No participants had to be excluded.

At T1, 271 adults participated, and 231 adults participated at T2 (attrition rate = 14.8%). Of the final sample, 118 participants were younger adults (*M*_age_ = 24.64, SD_age_ = 3.90, 75.4% female, and 39.8% with college degrees) and 113 participants were older adults (*M*_age_ = 71.00, SD_age_ = 6.03, 69.7% female, and 42.2% with a college degree). Table S9 provides more details on the demographics.

Attrition analyses showed that individuals who did not participate in T2 had a significantly lower implicit self‐concept of emotional stability than participants who completed T2. There were no differences in other variables of interest (see Table S10 for more details).

#### 3.1.2 Procedure

##### 3.1.2.1 T1: Online Assessment

The participants received information about the study, gave informed consent, and chose an appointment for the laboratory session. Sixteen days before their appointment, they received an email with the link to the first online survey on SoSci Survey (Leiner 2021). Participants first generated a personalized code for data matching and then answered questions on demographic information and control variables relevant to the cardiac measurement. Last, we assessed explicit and implicit self‐concepts of the personality traits.

##### 3.1.2.2 T2: Laboratory Session

T2 took place 12–16 days after T1. In each session, two adults of the same age group participated. In cases where only one participant showed up (e.g., somebody canceled on short notice), a research assistant operated as a confederate (35% of sessions). Experimenters and confederates were psychology bachelor's or master's students who underwent extensive training with the first author before their first session with actual participants.

The laboratory session (T2) was piloted with six participants (four younger adults and two older adults) in February 2022. After participating, the pilot participants were interviewed, and none of them expressed concerns about the intensity of stress or the credibility of the experiment.

**FIGURE 2:**
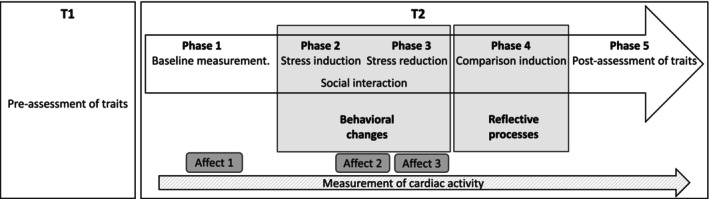
Schematic overview of the study procedure.

The main goal of T2 was to enact a TESSERA sequence for emotional stability and extraversion. For this purpose, the session consisted of four phases in which we elicited increased trait‐relevant states, induced past‐temporal or social comparisons, and, lastly, assessed personality traits. Figure 2 provides an overview of the procedure described next.

###### 3.1.2.2.1 Phase 1: Baseline heart rate measurement and questionnaires

At the start of each session, participants were seated at separate desks with computers. The experimenter explained the procedure and the application of the heart rate sensors. After the application of heart rate sensors, participants wore headphones, and a 3‐min baseline measurement of cardiac activity occurred while they watched a nature video and were instructed to sit as still as possible.

###### 3.1.2.2.2 Phase 2: Stress induction

During Phase 2, we aimed to create a trait‐relevant situation for state emotional stability. Drawing from prior literature, the stress‐induction paradigm encompassed three main characteristics of stressful situations: threat to desired goals, uncontrollability, and social threat (for an overview, see Dickerson and Kemeny 2004). We combined different sources of stress while keeping their intensity at a medium level to simulate everyday stressors. Participants did a timed cognitive test (digit‐symbol test; Schupp et al. 2008) with the incentive of a bonus of up to €3 depending on their performance. The goal to master the test was threatened when, just before its completion, a programmed Windows error message appeared, and the browser closed abruptly, leaving participants with no control over the outcome (see Riedl et al. 2012). As foreseen, all participants then informed the experimenter about the browser crash. To create a social threat, the experimenter initially reacted in a confused and stressed manner. After both participants encountered the “same problem,” the experimenter instructed the participants to proceed to the next task while allegedly attempting to find out whether the data were saved and to resolve the technical problem.

###### 3.1.2.2.3 Phase 3: Stress reduction

In Phase 3, we explicitly instructed the participants to act very extraverted and emotionally stable in a conversation with the other participant. This was tested in previous research and predicted increases in positive and decreases in negative affect (McNiel and Fleeson 2006). Being talkative is one of the main characteristics of extraversion (Soto and John 2017), and talking to strangers is associated with lowered shyness (Sandstrom and Boothby 2021). In addition, research showed that thinking about positive experiences reduces stress reactions (Speer and Delgado 2017). Guided by this literature, the participants were instructed to think about an experience they found pleasant within the last 4 weeks and to tell this experience to the other participant. Participants took turns. They were also instructed to behave as sociable and relaxed as possible, smile, and ask the other person one or more questions about the event. By this, we ensured that the reactions of the interaction partners were positive and attentive. Accordingly, increased positive affect and feedback should also enable the positive reinforcement of increased states of emotional stability and extraversion (Gawronski and Bodenhausen 2006; Wrzus and Roberts 2017). Participants evaluated both their own behavior and that of the other participant during this phase. Details on the measures used are provided in section 3.1.3.

###### 3.1.2.2.4 Phase 4: Past‐temporal versus social comparisons and personality assessment

After concluding the social interaction task, the experimenter informed the participants of the successful recovery of their data and that they could return to finish the interrupted survey. The purpose of Phase 4 was to induce a downward past‐temporal versus social comparison to which participants were randomly assigned. In the social comparison condition, participants were informed per text and graph that other people in the current study had an average mean of 2.9 on extraversion and 2.8 on emotional stability (i.e., slightly below average on a scale from 1 to 5). In the past‐temporal comparison condition, the same values were given as their values in the survey 2 weeks prior. As participants behaved very extraverted and emotionally stable in the previous conversation situation, this manipulation was intended to induce a downward comparison (i.e., the realization that they were more extraverted or emotionally stable than before or compared to others). Afterwards, the explicit and implicit personality trait self‐concepts were assessed, and participants responded to the manipulation checks (see Section 3.1.3). Participants were debriefed online 2 weeks later. Three of them (< 1%) reported that they were suspicious about the stress induction.

#### 3.1.3 Measures

##### 3.1.3.1 Explicit Self‐Concepts

These were measured as in Study 1 (BFI‐2; Danner et al. 2019; Soto and John 2017). Internal consistency estimates of emotional stability (ω_T1_ = 0.87, range ω_T1_: 0.85–0.90; ω_T2_ = 0.88, range ω_T2_: 0.86–0.90) and extraversion (ω_T1_ = 0.86, range ω_T1_: 0.84–0.89; ω_T2_ = 0.88, range ω_T2_: 0.85–0.90) were excellent.

##### 3.1.3.2 Implicit Self‐Concepts

The assessment of implicit self‐concepts and the IAT scoring algorithm was identical to Study 1 (Greenwald et al. 2003; Richetin et al. 2015). Split‐half reliabilities were acceptable for both traits, with an internal consistency of 0.76 (T1) and 0.74 (T2) for emotional stability and 0.92 (T1) and 0.89 (T2) for extraversion.

##### 3.1.3.3 Affect, Cardiovascular Activity, Behavioral Ratings, and Comparisons as Manipulation Checks

We implemented manipulation checks to ensure that the experiment had the intended effects. Accordingly, we analyzed components of the TESSERA sequence (states/state expressions, reactions) and reflective processes represented by changes in affect and cardiac activity, states, and the comparison standards employed.

State affect was measured after the baseline measurement in Phase 1, after the stress induction at the beginning of Phase 3, and after the social stress reduction at the end of Phase 3 (Figure 2). Six bipolar item pairs, scaled from 1 to 7, measured positive versus negative affect and arousal (e.g., “stressed” vs. “relaxed”). Items were adapted from the Multidimensional Mood Questionnaire (Hinz et al. 2012). Affect was calculated as the mean of the individual values of all item pairs. Internal consistency was excellent, with an average of ω = 0.92 and a range of ω = 0.88–0.94. Additionally, cardiovascular activity, which changes quickly under stress, was assessed with a heart rate sensor with adhesive electrodes that were applied by the participants to their chest (Movisens Gmbh n.d.).

Using the software DataAnalyzer *Version 1.13.5* (Movisens GmbH 2019), we calculated the average heart rate and the RMSSD (Malik 1996) of each phase, which are suitable indicators of sympathetic (heart rate) and parasympathetic control (heart rate and RMSSD) for short‐term measurements (Denver et al. 2007; Malik 1996). Higher heart rates and lower RMSSD values are considered indicators of stress (Denver et al. 2007; Malik 1996). Depending on the experimental phase, *n* = 43–53 mean values were missing for the heart rate, and *n* = 82–90 mean values were missing for the RMSSD due to insufficient quality of cardiac data. Potential reasons are the incorrect application of sensors, body composition, sweat, or movement artifacts (Cosoli et al. 2020; Hernández‐Vicente et al. 2021).

To check whether the behavioral manipulation was successful, the participants rated themselves and the other participant on their behavior during the social interaction/stress reduction. Five bipolar item pairs (adapted from Breil et al. 2022; McNiel and Fleeson 2006; Schmukle et al. 2008), measured state emotional stability (three items, e.g., “stressed” vs. “relaxed”) and extraversion (two items, e.g., “shy” vs. “talkative”), scaled from 1 to 7 (average ω = 0.93 for emotional stability, average ω = 0.89 for extraversion.).

After the assessment of personality traits, we included a manipulation check regarding a past‐temporal comparison (“I thought about how my experiences and behaviors are today compared to when I was first surveyed”); social comparison (“I thought about how my experience and behavior compared to other people”); and a reflection on recent behavior (“I thought about how I behaved today”). There were also two filler items (e.g., “I chose my answers intuitively”). The participants responded on a 5‐point Likert scale from 1 (*disagree strongly*) to 5 (*agree strongly*).

#### 3.1.4 Analytic Strategy

We winsorized the values of all variables (*M* ± 3 SD) in cases of outliers (*n* = 4). Age was coded as −1 = younger adults (18–33 years) and 1 = older adults (60+ years) based on the bimodal distribution of the variable. Experimental conditions were coded as −1 = social comparison, 1 = past‐temporal comparison. We used the same latent change analyses as in Study 1, with the only difference that we entered all manifest predictors into one model, predicting change in latent explicit or implicit self‐concepts: age, the comparison condition, and the interaction between age and the comparison condition (see Figure S1). Again, all models were defined with invariant factor loadings and intercepts, as strong measurement invariance was tested and held in each measurement model (Chen 2007; see Table S11 for details). To test the robustness of effects, we additionally performed all analyses controlling for whether a confederate (coded as 1) versus another participant (coded as −1) was present.

### 3.2 Results

Altogether, 121 participants (66 younger and 55 older adults) were randomly assigned to the past‐temporal comparison condition, and 110 (52 younger and 58 older adults) to the social comparison condition. Table 4 displays descriptive information on the explicit and implicit self‐concepts of emotional stability and extraversion at T1 and T2.

**TABLE 4:** Descriptive statistics and correlations of variables of interest.

	1	2	3	4	5	6	7	8	9
1. ES explicit T1									
2. ES explicit T2	**0.90**								
3. ES implicit T1	0.27	0.21							
4. ES implicit T2	0.12	0.11	**0.44**						
5. EX explicit T1	0.21	0.16	0.16	0.09					
6. EX explicit T2	0.23	0.21	0.13	0.03	**0.91**				
7. EX implicit T1	0.21	0.15	0.14	0.08	0.40	0.41			
8. EX implicit T2	0.25	0.21	0.17	0.14	0.41	0.44	**0.58**		
9. Age	0.35	0.32	0.27	0.16	0.12	0.12	0.19	0.25	
*M* (SD)	3.29 (0.66)	3.34 (0.66)	0.39 (0.36)	0.34 (0.32)	3.34 (0.64)	3.37 (0.65)	0.01 (0.61)	0.03 (0.56)	47.31 (23.76)

*Note:* All correlation coefficients larger than |0.19| are significant with *p* < 0.05. Rank‐order correlations are bolded.

Abbreviations: ES, emotional stability; EX, extraversion.

#### 3.2.1 Manipulation Checks

**FIGURE 3:**
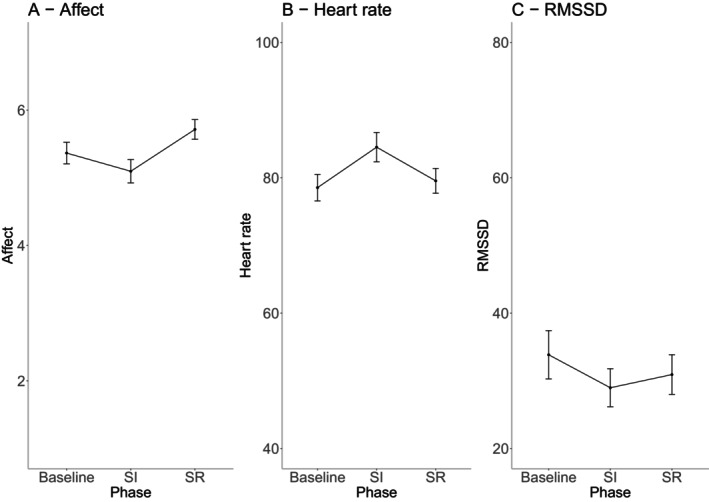
Change of affect (A), heart rate (B), and RMSSD (C) across the phases of the experiment. SI = stress induction, SR = stress reduction. Error bars represent confidence intervals of the mean. Affect ratings represent the mean and were assessed before the baseline and after the stress induction and reduction, respectively. Parameters of cardiac activity were assessed continuously; data points represent the mean of each phase.

The manipulation checks showed that the experiment affected participants as intended: While affective well‐being and heart rate variability (RMSSD) significantly decreased during stress induction and increased during stress reduction, the heart rate showed the opposite trend (see Figure 3). Two‐sided *t*‐tests showed no significant differences in affect and cardiac activity between comparison conditions (see Table S12). Further, mixed‐effects ANOVAs (baseline vs. stress induction, stress induction vs. stress reduction) demonstrated significant main effects of the experimental phases and age (see Table S13 for details). Interaction effects of experimental phase and age occurred only in the transition from stress induction to reduction (not from baseline to stress induction) and for both cardiac parameters. Pairwise comparisons with Bonferroni correction indicated that the heart rate significantly decreased during the stress reduction phase for both age groups, with a more pronounced effect in younger adults (see Figure S2, Panel B). RMSSD increased during stress reduction for younger adults, but not for older adults (see Figure S2, Panel C). These findings align with previous research, suggesting that affect increases and recovers similarly in younger and older adults after a stressor, while cardiac activity recovers more slowly in older adults (Wrzus et al. 2014).

After the stress reduction, participants of both experimental conditions reported highly emotionally stable (*M* = 5.63, SD = 1.25) and extraverted (*M* = 5.87, SD = 1.23) behavior for themselves and their interaction partners (ES: *M* = 5.86, SD = 1.15, EX: *M* = 5.93, SD = 1.16; see Table S12), which were comparable to those reported in previous experimental studies (Gallagher et al. 2011; McNiel and Fleeson 2006). Also, the participants reported that they experienced the social interaction very positively (M = 6.11, SD = 1.13, range 1–7) and not as exhausting (*M* = 2.26, SD = 1.56, range 1–7).

After the comparison induction and personality assessment, the participants completed the manipulation check on comparisons during their self‐reports. There was no significant difference in considering past‐temporal or social comparisons, nor how much they reflected on the behavior of that day between the experimental conditions (see Table 5). Younger and older adults did not differ in considering their scores from 2 weeks prior. However, younger adults reported significantly more consideration of other people's standing on these traits (Cohen's *d* = 0.55) and their behavior during the same day (Cohen's *d* = 0.33).

**TABLE 5:** Descriptives of self‐reflections and results of two‐sided *t*‐tests for independent samples.

Variables *M* (SD)	Experimental condition		Age group	
	PTCOMP	SCOMP	Younger adults	Older adults
Reported PTCOMP	2.12_a_ (1.28)	2.21_a_ (1.27)	2.19_a_ (1.27)	2.14_a_ (1.29)
Reported SCOMP	3.13_a_ (1.23)	3.28_a_ (1.31)	3.53_a_ (1.17)	2.85_b_ (1.30)
Reported reflection on behavior	2.46_a_ (1.25)	2.39_a_ (1.31)	2.61_a_ (1.29)	2.19_b_ (1.22)

*Note:* Means with different subscripts differ significantly between conditions/age groups with *p* < 0.05.

Abbreviations: PTCOMP, past‐temporal comparison; SCOMP, social comparison.

#### 3.2.2 Results of Hypotheses Testing

The results of the latent change analyses are displayed in Table 6. We specified one model per trait and measure that included all variables simultaneously to reduce the number of tests. Table 7 provides model fit indices of the final models. In H1a, we stated that changes in extraversion should be more pronounced than changes in emotional stability. However, the analyses provided evidence that this was not the case: The explicit self‐concepts of both traits increased, while the implicit self‐concepts of both traits remained the same. Regarding H1b, we found partial support. Changes in extraversion were not more pronounced when participants compared themselves to others. However, as expected, there was also no difference in the change of emotional stability between the two comparison conditions. Supporting H2a, the explicit self‐concepts of both traits increased, while the implicit self‐concepts did not. Furthermore, as hypothesized (H2b), the implicit self‐concepts were not affected by the comparison condition. Contrary to our assumptions (H3a), personality changes were not less pronounced among older than among younger adults. In H3b, we stated that younger adults' self‐concepts would change more in the social comparison condition, while older adults' self‐concepts would change more in the past‐temporal comparison condition. Again, our results did not support this hypothesis.

**TABLE 6:** Past‐temporal versus social comparisons, age, and comparison by age interactions predicting short‐term changes in the explicit and implicit self‐concepts of emotional stability and extraversion.

Model	Emotional stability		Extraversion	
	Estimate [95% CI]	*p*	Estimate [95% CI]	*p*
**Explicit**				
Change T2–T1	0.057 [0.020, ∞]	**0.006**	0.047 [0.009, ∞]	**0.022**
COMP	0.023_a_ [−0.020, 0.066]	0.298	−0.015 [−∞, 0.021]	0.245
Age	−0.020 [−∞, 0.018]	0.193	0.014 [−∞, 0.052]	0.281
COMP by Age	−0.018 [−0.055, ∞]	0.219	0.015 [−0.022, ∞]	0.248
**Implicit**				
Change T2–T1	−0.041 [−0.081, ∞]	0.046	0.018 [−0.040, ∞]	0.306
COMP	−0.003_a_ [−0.039, 0.034]	0.875	0.011_a_ [−0.048, 0.070]	0.711
Age	−0.024 [−∞, 0.015]	0.160	0.018 [−∞, 0.075]	0.304
COMP by Age	−0.004_a_ [−0.040, 0.032]	0.832	−0.009_a_ [−0.068, 0.049]	0.760

*Note:* Experimental conditions were coded as −1 = social comparison, 1 = past‐temporal comparison. Age was coded as −1 = younger adults and 1 = older adults. Significant *p* values (*p* < 0.05) are bolded. _a_Undirected hypothesis.

Abbreviation: COMP, comparison condition.

**TABLE 7:** Model fit indices predicting short‐term change in the explicit and implicit self‐concepts of emotional stability and extraversion.

Model	Χ^2^	CFI	TLI	RMSEA	SRMR
ES explicit	21.960	1.000	1.000	0.000	0.043
EX explicit	25.833	0.997	0.996	0.023	0.039
ES implicit	5.925	1.000	1.000	0.000	0.025
EX implicit	14.572	0.995	0.991	0.037	0.047

Abbreviations: CFI, comparative fit index; ES, emotional stability; EX, extraversion; RMSEA, root mean square error of approximation; SRMR, standardized root mean square residual; TLI, Tucker–Lewis index.

Robustness checks examining whether the effects depended on the presence of a confederate or another participant during the experiment indicated that the results were not influenced by the type of interaction partner (see Table S14).

## 4 Discussion

In this research, we aimed to examine whether past‐temporal and social comparisons have different effects on changes in the explicit and implicit self‐concepts of emotional stability and extraversion at different ages. We explored our research questions with two multi‐method studies with longitudinal and experimental designs.

### 4.1 The Effects of Comparison Standards on Changes in the Explicit Self‐Concept

Prior research had proposed that reflective processes, such as past‐temporal and social comparisons, translate everyday experiences into long‐term personality change (Jackson and Wright 2024; Morina 2021; Wrzus and Roberts 2017), yet no empirical studies had tested this assumption. On average, no mean‐level trait change occurred across 6 months in longitudinal Study 1, which is in line with previous findings over similar timespans (Quintus et al. 2021). Considering individual differences in trait change, individuals who compared themselves more frequently with themselves in the past or to others increased more strongly in emotional stability. Conversely, the frequency of both types of comparisons regarding extraversion was not linked to individual differences in trait changes, potentially because extraversion changes less strongly across the lifespan (Bleidorn et al. 2022). Additionally, the direction of comparisons could vary more often for extraversion because, as an interpersonal trait, it may depend more on others' trait levels, particularly during social comparisons. Alternating comparisons with less and more extraverted individuals could cancel out effects. Of course, such a phenomenon could also have dampened the effects concerning emotional stability, which consequently might have been underestimated. Indeed, it appears essential to control the direction of comparisons. While the design of Study 1 did not allow for control of the direction, a downward comparison was induced in experimental Study 2: Participants acted emotionally stable and extraverted after a stressful situation and were then instructed to compare themselves to their somewhat lower scores 2 weeks before or to others with lower scores. Across this short intervention, the explicit self‐concepts of emotional stability and extraversion increased. This result provides initial evidence for theoretical assumptions (Baumert et al. 2017; Jackson and Wright 2024; Wrzus and Roberts 2017) that the combination of trait‐relevant state changes and self‐reflections shows effects on explicit self‐concepts. Further, these findings of Study 2 cohere with cross‐sectional findings on the effects of past‐temporal comparisons on extraversion (Hanko et al. 2010).

As predicted and aligned with the Study 1 results, the increase in emotional stability did not differ between comparison conditions in Study 2. The same was true for extraversion, meaning that contrary to our predictions, social comparisons did not enhance change in the explicit self‐concept of extraversion more than past‐temporal comparisons. Overall, these findings suggest that both types of comparisons were similarly effective regarding changes in explicit self‐concepts, which could be because the discrepancy between the individual's behavior with their scores two weeks prior or with others could have been similarly apparent. Both types of comparisons could be common underlying mechanisms of change in self‐concepts, as they complement each other due to co‐occurrence (Möller and Marsh 2013). In line with this, Study 1 showed a high correlation between past‐temporal and social comparisons for both traits, and Study 2 showed a co‐occurrence of both comparison types across conditions (e.g., participants in the past‐temporal condition also engaged in social comparisons, and vice versa). Also, in domains other than personality, both comparisons had similar effects on short‐term self‐concept change (academic self‐concept: Wolff et al. 2018; task performance: Zell and Alicke 2009).

The change in the explicit self‐concept of emotional stability was influenced by comparisons both long‐ and short‐term. In contrast, the self‐concept of extraversion was affected only short‐term. This suggests that extraversion may change immediately after downward comparisons and could tend to regress to its set‐point level more easily over time (Ormel et al. 2017). Indeed, longitudinal intervention studies showed that emotional stability increases more strongly than extraversion (Roberts et al. 2017; Stieger et al. 2021) and therefore might be more malleable.

Exploratory analyses of Study 1 demonstrated the robustness of the effects: The findings were consistent across countries, and the positive effect of comparisons on changes in the explicit self‐concept of emotional stability persisted even when controlling for general self‐reflection tendencies. Moreover, these results supported the assumption that self‐reflections need to be assessed in a trait‐specific manner to accurately predict trait changes.

To conclude, the results of our studies give first evidence that downward past‐temporal and social comparisons can enhance explicit trait self‐concepts when combined with behavioral changes. While the findings of the experimental Study 2 may reflect short‐term changes, they could represent the process of gradual shifts in explicit self‐concepts: Using the same trait measure, after just one brief behavioral change intervention, we found more pronounced change within explicit self‐concepts than across 6 months. Therefore, our findings align with theoretical frameworks stating that reflective processes translate behavioral manifestations into changes in explicit self‐concepts (Baumert et al. 2017; Morina 2021; Wrzus and Roberts 2017).

### 4.2 Changes in the Explicit Versus Implicit Self‐Concepts

Explicit and implicit self‐concepts are related yet distinct entities that presumably form and develop through partly different processes: reflective versus associative. Based on theory (Wrzus and Roberts 2017), we tested whether past‐temporal comparisons also enhance changes in implicit self‐concepts by reinforcing repetitions of previously shown behavioral patterns. The results of Study 1, however, showed that this was not the case for the implicit self‐concepts of emotional stability or extraversion. As expected, the frequency of social comparisons also did not affect changes in the implicit representations of both traits. Also, in Study 2, there was no difference in the change of implicit self‐concepts between comparison conditions nor a significant change in extraversion across conditions. Regarding emotional stability, the change in the implicit self‐concept did not differ between conditions, and, contrary to our hypothesis that the implicit self‐concept would increase, it decreased in both comparison conditions. As the effects are in the opposite direction of hypothesized, they should be taken cautiously, and we refrain from further interpretations (Cho and Abe 2013). Nevertheless, we note that divergent changes in the explicit and implicit self‐concepts have also been observed in previous research (Wrzus et al. 2023). Our results partially support theoretical assumptions about different processes involved in changing explicit and implicit self‐concepts (Gawronski and Bodenhausen 2006; Wrzus and Roberts 2017), and prior evidence that reflections do not affect the implicit self‐concept (Gawronski and Bodenhausen 2006; Quintus et al. 2021). The experiment successfully induced trait‐relevant behavior and positive affect, aiming to reinforce the behavior with positive feedback. However, most likely changing associative patterns may take repeated behavioral change instead of one incident (cf. Gawronski and Bodenhausen 2006; Wrzus and Roberts 2017).

### 4.3 Age Differences in Personality Processes

We proposed that age differences in trait change may stem from reflective and associative processes being less effective with older individuals compared to younger individuals. In Study 1, we did not find evidence for age differences in the change of explicit or implicit self‐concepts, which contradicts previous research regarding changes in explicit self‐concepts (Bleidorn et al. 2022). While in general the extent of personality change may vary by age (Bleidorn et al. 2022), a longer period of observation might be required to capture these differences. Exploratory cross‐country analyses revealed that within the US sample, the implicit self‐concept changed more strongly in younger individuals than in older individuals. However, this age difference was not apparent in Germany. Although overall the age effect in the United States was in line with our hypotheses, no theoretical background or previous evidence supports the country difference related to this finding. Importantly, most studies on country differences were cross‐sectional and lacked implicit measures (e.g., McCrae et al. 1999). One longitudinal study found a stronger decline in the explicit self‐concept of extraversion across the lifespan in the United States compared to Japan (Chopik and Kitayama 2018), suggesting potential cross‐country differences. However, differences in the current study might reflect sampling or contextual effects related to the assessment period. Future research could examine such cross‐country differences in more detail with implicit measures.

Contrary to our predictions, in Study 2, younger and older adults adapted their explicit self‐concepts of both traits similarly. Thus, although older adults use trait‐specific comparisons less frequently (Küchler et al. 2025), they may adapt their self‐concepts once confronted with them. Further, in Study 2, we found age differences in the information considered when completing a self‐report on their personality: Older adults reported significantly less consideration of social comparisons and current behavior during their personality assessment. Hence, similarities in short‐term effects might diminish over time as older adults might reject new information about themselves (Gawronski and Bodenhausen 2006) as they believe to know themselves better (Diehl and Hay 2011).

### 4.4 Limitations and Future Directions

Despite the strengths of using a multi‐method approach, some limitations need to be considered. First, our study was limited to emotional stability and extraversion. As the predictions about how reflective processes shape personality development were based on general theoretical frameworks (Baumert et al. 2017; Jackson and Wright 2024; TESSERA, Wrzus and Roberts 2017), we propose that our findings could apply to other traits as well. Initial evidence stems from long‐term personality interventions, combining behavioral and reflective tasks for other Big Five traits (Stieger et al. 2021), social–emotional well‐being (Pollock et al. 2023), and intellectual humility (Mendonça et al. 2023).

Second, we focused on past‐temporal and social comparisons because they are employed most often (Morina 2021). However, different types of self‐reflections may influence each other, and past‐temporal and social comparisons likely often happen simultaneously (Möller and Marsh 2013), as shown by the manipulation checks and high associations in both of our studies. Thus, even when a person perceives their behavior as more extraverted than usual (past‐temporal comparison), comparing themselves to someone more extraverted (social comparison) could reduce the effect of the past‐temporal comparison.

Third, in Study 2, effects between comparison conditions were indistinguishable, and no control group without comparison induction existed. Thus, trait changes might have resulted solely from the induced behavior. Still, only explicit, not implicit, self‐concepts increased, aligning with theories that behavioral change alone may not suffice for explicit self‐concept change (Gawronski and Bodenhausen 2006; Wrzus and Roberts 2017). We anticipated that participants would naturally engage in downward comparisons. This expectation was based on their stable and extraverted behavior in the previous experimental phase (i.e., behavioral induction during the conversation), contrasted with below‐average values of the traits presented in the comparison induction. However, we did not include a manipulation check regarding the comparison direction (upward, downward, lateral) and did not provide explicit feedback, as this could have created strong demand effects and would have been less representative of real‐life situations. Therefore, future research should include no‐comparison versus upward versus downward conditions to clarify these findings, which were not feasible in this research due to time and financial constraints.

Fourth, typical of research that investigates desirable change, we cannot completely rule out demand effects. Importantly, our focus was on the fundamental processes of trait change, relevant regardless of whether individuals intend to change, as in daily life. Thus, our sample did not comprise participants actively seeking change or with particularly low trait levels.

Fifth, the short time scale may raise the question of whether the observed effects represent long‐term trait changes. Importantly, studies with longer durations cannot capture short‐term processes. While Study 1 showed effects of comparisons over six months, Study 2 focused on temporary increases in trait levels. These findings suggest that experiences combined with comparisons may lead to long‐term change (Study 1). Additionally, although these changes are theorized to be stronger when repeated experiences and reflections occur (Wrzus and Roberts 2017), the short‐term mechanism could be observed after one single experience (Study 2). We encourage future research to test our hypotheses with repeated measurements over longer time frames.

Finally, our samples consisted of volunteers from the general population. Study 1 was socio‐demographically diverse but had a large attrition rate in the US subsample, with non‐completers showing slightly lower scores on some variables. Study 2 mostly included individuals with high socioeconomic status, which is linked to better health with older age (see Wagg et al. 2021), and all participants were able to concentrate and attend the lab‐based experiment online sessions. Therefore, the findings may not be fully generalizable to a more diverse population of older adults.

## 5 Conclusion

While several previous intervention studies on processes of personality development have focused on pre‐behavioral factors and behavioral changes, the current research additionally emphasizes comparisons as reflective, post‐behavioral processes for change in the social–emotional traits of emotional stability and extraversion. In line with theoretical considerations and previous work on attitude change (Gawronski and Bodenhausen 2006; Gawronski and LeBel 2008), we also provided evidence that changes in implicit trait self‐concepts are not (substantially) affected by reflective processes. Although we did not observe age differences in the effects of reflective and associative processes, we found older adults to focus less on social comparisons and recent behavior when evaluating their standing on personality traits. Innovatively, we changed relatively stable trait self‐concepts with a single brief behavioral induction, further substantiating the relevance of behavioral changes theorized within personality development frameworks (Baumert et al. 2017; Jackson and Wright 2024; Wrzus and Roberts 2017). Future research could expand the current findings regarding further and narrower traits, additional reflective processes, and larger time spans. Given the significant impact of social–emotional traits on mental health (Lamers et al. 2012), professional success, and personal achievements (Roberts et al. 2007), understanding the processes that facilitate or hinder personality change in these traits is crucial when applying the findings in coaching, educational, and therapeutic programs.

## Author Contributions

All authors: Conceptualization, Methodology **Gabriela Küchler:** investigation, software, data curation and preparation, formal analysis, writing – original draft preparation, visualization; **Kira S. A. Borgdorf:** investigation, data curation, writing – reviewing and editing; **Corina Aguilar‐Raab:** project administration, funding acquisition, supervision, writing – reviewing and editing; **Cornelia Wrzus:** project administration, funding acquisition, supervision, writing – reviewing and editing.

## Ethics Statement

The Ethics Committee of the Psychological Institute of the Heidelberg University approved the research project.

## Conflicts of Interest

The authors declare no conflicts of interest.

## Supporting information


Appendix S1

## Data Availability

Data, code, and materials of this study can be accessed from https://osf.io/rwkjf/.

## Endnotes

^1^
As the original scoring key (Soto and John 2017) was set for negative emotionality/neuroticism, we coded the items in the opposite direction compared to the original literature. That is, items that were reverse‐coded in the original were not reverse‐coded in the current study, and vice versa. See the R script for more details.

^2^
Initially, we chose an age range of 18–33 and 65–80 to ensure equal age spans for younger and older adulthood. Later on, due to difficulties in recruiting older participants, we expanded the older group to include those aged 60+.

## References

[jopy13016-bib-0001] Alessandri, G. , A. Zuffianò , M. Vecchione , B. M. Donnellan , and J. Tisak . 2016. “Evaluating the Temporal Structure and Correlates of Daily Self‐Esteem Using a Trait State Error Framework (TSE).” Self and Identity 15, no. 4: 394–412. 10.1080/15298868.2015.1137223.

[jopy13016-bib-0002] Back, M. D. , S. C. Schmukle , and B. Egloff . 2009. “Predicting Actual Behavior From the Explicit and Implicit Self‐Concept of Personality.” Journal of Personality and Social Psychology 97, no. 3: 533–548. 10.1037/a0016229. 19686006

[jopy13016-bib-0003] Baltes, P. B. 1987. “Theoretical Propositions of Life‐Span Developmental Psychology: On the Dynamics Between Growth and Decline.” Developmental Psychology 23, no. 5: 611–626. 10.1037/0012-1649.23.5.611.

[jopy13016-bib-0004] Baumert, A. , M. Schmitt , M. Perugini , et al. 2017. “Integrating Personality Structure, Personality Process, and Personality Development.” European Journal of Personality 31, no. 5: 503–528. 10.1002/per.2115.

[jopy13016-bib-0005] Bishop, N. A. , T. Lu , and B. A. Yankner . 2010. “Neural Mechanisms of Ageing and Cognitive Decline.” Nature 464, no. 7288: 529–535. 10.1038/nature08983. 20336135 PMC2927852

[jopy13016-bib-0006] Bleidorn, W. , and C. J. Hopwood . 2024. “A Motivational Framework of Personality Development in Late Adulthood.” Current Opinion in Psychology 55: 101731. 10.1016/j.copsyc.2023.101731. 38007918

[jopy13016-bib-0007] Bleidorn, W. , T. Schwaba , A. Zheng , et al. 2022. “Personality Stability and Change: A Meta‐Analysis of Longitudinal Studies.” Psychological Bulletin 148, no. 7–8: 588–619. 10.1037/bul0000365. 35834197

[jopy13016-bib-0008] Borgdorf, K. S. A. , G. Kuechler , C. Wrzus , and C. Aguilar‐Raab . 2024. “Less Frequent but Equally Useful: Social and Temporal Comparisons in Light of Mindfulness and Self‐Compassion.” Mindfulness 15, no. 11: 2906–2918. 10.1007/s12671-024-02472-w.

[jopy13016-bib-0009] Breil, S. M. , P. C. Schweppe , K. Geukes , et al. 2022. “The Incremental Validity of Average States: A Replication and Extension of Finnigan and Vazire (2018).” Journal of Personality and Social Psychology 123, no. 3: e23–e37. 10.1037/pspp0000408. 35113627

[jopy13016-bib-0010] Bühler, J. L. , U. Orth , W. Bleidorn , et al. 2024. “Life Events and Personality Change: A Systematic Review and Meta‐Analysis.” European Journal of Personality 38, no. 3: 544–568. 10.1177/08902070231190219.

[jopy13016-bib-0011] Buunk, A. P. , J. Cohen‐Schotanus , and R. H. Van Nek . 2007. “Why and How People Engage in Social Comparison While Learning Social Skills in Groups.” Group Dynamics: Theory, Research, and Practice 11, no. 3: 140–152. 10.1037/1089-2699.11.3.140.

[jopy13016-bib-0012] Callan, M. J. , H. Kim , and W. J. Matthews . 2015. “Age Differences in Social Comparison Tendency and Personal Relative Deprivation.” Personality and Individual Differences 87: 196–199. 10.1016/j.paid.2015.08.003.

[jopy13016-bib-0013] Carstensen, L. L. , D. M. Isaacowitz , and S. T. Charles . 1999. “Taking Time Seriously: A Theory of Socioemotional Selectivity.” American Psychologist 54, no. 3: 165–181. 10.1037/0003-066X.54.3.165. 10199217

[jopy13016-bib-0014] Caspi, A. , and B. W. Roberts . 2001. “Personality Development Across the Life Course: The Argument for Change and Continuity.” Psychological Inquiry 12, no. 2: 49–66. 10.1207/S15327965PLI1202_01.

[jopy13016-bib-0015] Charlesworth, T. E. S. , and M. R. Banaji . 2022. “Patterns of Implicit and Explicit Attitudes: IV. Change and Stability From 2007 to 2020.” Psychological Science 33, no. 9: 1347–1371. 10.1177/09567976221084257. 35895290

[jopy13016-bib-0016] Chen, F. F. 2007. “Sensitivity of Goodness of Fit Indexes to Lack of Measurement Invariance.” Structural Equation Modeling: A Multidisciplinary Journal 14, no. 3: 464–504. 10.1080/10705510701301834.

[jopy13016-bib-0017] Cho, H.‐C. , and S. Abe . 2013. “Is Two‐Tailed Testing for Directional Research Hypotheses Tests Legitimate?” Journal of Business Research 66, no. 9: 1261–1266. 10.1016/j.jbusres.2012.02.023.

[jopy13016-bib-0018] Chopik, W. J. , and S. Kitayama . 2018. “Personality Change Across the Life Span: Insights From a Cross‐Cultural, Longitudinal Study.” Journal of Personality 86, no. 3: 508–521. 28646503 10.1111/jopy.12332 PMC5742083

[jopy13016-bib-0019] Cosoli, G. , S. Spinsante , and L. Scalise . 2020. “Wrist‐Worn and Chest‐Strap Wearable Devices: Systematic Review on Accuracy and Metrological Characteristics.” Measurement 159: 107789. 10.1016/j.measurement.2020.107789.

[jopy13016-bib-0020] Costa, P. T. , and R. McCrae . 1989. “The Structure of Interpersonal Traits: Wiggins's Circumplex and the Five‐Factor Model.” Journal of Personality and Social Psychology 56, no. 4: 586–595. 10.1037/0022-3514.56.4.586. 2709308

[jopy13016-bib-0021] Craik, F. I. M. , and E. Bialystok . 2006. “Cognition Through the Lifespan: Mechanisms of Change.” Trends in Cognitive Sciences 10, no. 3: 131–138. 10.1016/j.tics.2006.01.007. 16460992

[jopy13016-bib-0022] Crescentini, C. , C. Urgesi , F. Campanella , R. Eleopra , and F. Fabbro . 2014. “Effects of an 8‐Week Meditation Program on the Implicit and Explicit Attitudes Toward Religious/Spiritual Self‐Representations.” Consciousness and Cognition 30: 266–280. 10.1016/j.concog.2014.09.013. 25441977

[jopy13016-bib-0023] Cutler, J. , M. K. Wittmann , A. Abdurahman , et al. 2021. “Ageing Is Associated With Disrupted Reinforcement Learning Whilst Learning to Help Others Is Preserved.” Nature Communications 12, no. 1: 4440. 10.1038/s41467-021-24576-w. PMC8295324 34290236

[jopy13016-bib-0024] Danner, D. , B. Rammstedt , M. Bluemke , et al. 2019. “Das Big Five Inventar 2 [The German Big Five Inventory 2: Measuring Five Personality Domains and 15 Facets].” Diagnostica 65, no. 3: 121–132. 10.1026/0012-1924/a000218.

[jopy13016-bib-0025] Denver, J. W. , S. F. Reed , and S. W. Porges . 2007. “Methodological Issues in the Quantification of Respiratory Sinus Arrhythmia.” Biological Psychology 74, no. 2: 286–294. 10.1016/j.biopsycho.2005.09.005. 17067734 PMC1828207

[jopy13016-bib-0026] Dickerson, S. S. , and M. E. Kemeny . 2004. “Acute Stressors and Cortisol Responses: A Theoretical Integration and Synthesis of Laboratory Research.” Psychological Bulletin 130, no. 3: 355–391. 10.1037/0033-2909.130.3.355. 15122924

[jopy13016-bib-0027] Diehl, M. , and E. L. Hay . 2011. “Self‐Concept Differentiation and Self‐Concept Clarity Across Adulthood: Associations With Age and Psychological Well‐Being.” International Journal of Aging and Human Development 73, no. 2: 125–152. 10.2190/AG.73.2.b. 22010361 PMC3198817

[jopy13016-bib-0028] Egloff, B. , F. Weck , and S. C. Schmukle . 2008. “Thinking About Anxiety Moderates the Relationship Between Implicit and Explicit Anxiety Measures.” Journal of Research in Personality 42, no. 3: 771–778. 10.1016/j.jrp.2007.08.005.

[jopy13016-bib-0029] Freund, A. M. , and J. O. Ritter . 2009. “Midlife Crisis: A Debate.” Gerontology 55, no. 5: 582–591. 10.1159/000227322. 19571526

[jopy13016-bib-0030] Gallagher, P. , W. Fleeson , and R. H. Hoyle . 2011. “A Self‐Regulatory Mechanism for Personality Trait Stability: Contra‐Trait Effort.” Social Psychological and Personality Science 2, no. 4: 335–342. 10.1177/1948550610390701.

[jopy13016-bib-0031] Gawronski, B. , and G. V. Bodenhausen . 2006. “Associative and Propositional Processes in Evaluation: An Integrative Review of Implicit and Explicit Attitude Change.” Psychological Bulletin 132, no. 5: 692–731. 10.1037/0033-2909.132.5.692. 16910748

[jopy13016-bib-0116] Gawronski, B. , and E. P. LeBel . 2008. “Understanding Patterns of Attitude Change: When Implicit Measures Show Change, but Explicit Measures Do Not.” Journal of Experimental Social Psychology 44, no. 5: 1355–1361. 10.1016/j.jesp.2008.04.005.

[jopy13016-bib-0032] Gawronski, B. , M. Morrison , C. E. Phills , and S. Galdi . 2017. “Temporal Stability of Implicit and Explicit Measures: A Longitudinal Analysis.” Personality and Social Psychology Bulletin 43, no. 3: 300–312. 10.1177/0146167216684131. 28903689

[jopy13016-bib-0033] Geiser, C. 2011. Datenanalyse mit Mplus [Data analysis with Mplus]. VS Verlag für Sozialwissenschaften [Publisher for Social Sciences].

[jopy13016-bib-0034] Gerber, J. P. , L. Wheeler , and J. Suls . 2018. “A Social Comparison Theory Meta‐Analysis 60+ Years on.” Psychological Bulletin 144, no. 2: 177–197. 10.1037/bul0000127. 29144145

[jopy13016-bib-0035] Geukes, K. , M. van Zalk , and M. D. Back . 2018. “Understanding Personality Development: An Integrative State Process Model.” International Journal of Behavioral Development 42, no. 1: 43–51. 10.1177/0165025416677847.

[jopy13016-bib-0036] Grant, A. M. , J. Franklin , and P. Langford . 2002. “The Self‐Reflection and Insight Scale: A New Measure of Private Self‐Consciousness.” Social Behavior and Personality: An International Journal 30, no. 8: 821–835. 10.2224/sbp.2002.30.8.821.

[jopy13016-bib-0037] Greenwald, A. G. , B. A. Nosek , and M. R. Banaji . 2003. “Understanding and Using the Implicit Association Test: I. An Improved Scoring Algorithm.” Journal of Personality and Social Psychology 85, no. 2: 197–216. 10.1037/0022-3514.85.2.197. 12916565

[jopy13016-bib-0038] Greenwald, A. G. , T. A. Poehlman , E. L. Uhlmann , and M. R. Banaji . 2009. “Understanding and Using the Implicit Association Test: III. Meta‐Analysis of Predictive Validity.” Journal of Personality and Social Psychology 97, no. 1: 17–41. 10.1037/a0015575. 19586237

[jopy13016-bib-0117] Gschwendner, T. , W. Hofmann , and M. Schmitt . 2006. “Moderatoren der Konsistenz implizit und explizit erfasster Einstellungen und Persönlichkeitsmerkmale.” Psychologische Rundschau 57, no. 1: 13–33. 10.1026/0033-3042.57.1.13.

[jopy13016-bib-0039] Gürel, Ç. , E. Brummelman , and G. Overbeek . 2022. “Proudly Moving Forward and Feeling Connected: Adolescents' Daily Temporal Comparisons Relate to a Desire for Growth and Sense of Relatedness.” Emotion 22, no. 6: 1224–1238. 10.1037/emo0000920. 33382322

[jopy13016-bib-0040] Hanko, K. , J. Crusius , and T. Mussweiler . 2010. “When I and Me Are Different: Assimilation and Contrast in Temporal Self‐Comparisons.” European Journal of Social Psychology 40, no. 1: 160–168. 10.1002/ejsp.625.

[jopy13016-bib-0041] Head, D. , K. M. Rodrigue , K. M. Kennedy , and N. Raz . 2008. “Neuroanatomical and Cognitive Mediators of Age‐Related Differences in Episodic Memory.” Neuropsychology 22, no. 4: 491–507. 10.1037/0894-4105.22.4.491. 18590361 PMC2688704

[jopy13016-bib-0042] Hernández‐Vicente, A. , D. Hernando , J. Marín‐Puyalto , et al. 2021. “Validity of the Polar H7 Heart Rate Sensor for Heart Rate Variability Analysis During Exercise in Different Age, Body Composition and Fitness Level Groups.” Sensors 21, no. 3: 902. 10.3390/31030902. 33572800 PMC7866245

[jopy13016-bib-0043] Higgins, E. T. 1987. “Self‐Discrepancy: A Theory Relating Self and Affect.” Psychological Review 94, no. 3: 319–340. 10.1037/0033-295X.94.3.319. 3615707

[jopy13016-bib-0044] Hinz, A. , I. Daig , K. Petrowski , and E. Brähler . 2012. “Die Stimmung in der Deutschen Bevölkerung: Referenzwerte Für den Mehrdimensionalen Befindlichkeitsfragebogen MDBF. The Mood of the German Population: Reference Values for the Multidimensional Mood Questionnaire MDBF.” PPmP – Psychotherapie, Psychosomatik, Medizinische Psychologie 62, no. 2: 52–57. 10.1055/s-0031-1297960. 22271232

[jopy13016-bib-0045] Hofmann, W. , B. Gawronski , T. Gschwendner , H. Le , and M. Schmitt . 2005. “A Meta‐Analysis on the Correlation Between the Implicit Association Test and Explicit Self‐Report Measures.” Personality and Social Psychology Bulletin 31, no. 10: 1369–1385. 10.1177/0146167205275613. 16143669

[jopy13016-bib-0046] Hofmann, W. , T. Gschwendner , and M. Schmitt . 2005. “On Implicit–Explicit Consistency: The Moderating Role of Individual Differences in Awareness and Adjustment.” European Journal of Personality 19, no. 1: 25–49. 10.1002/per.537.

[jopy13016-bib-0047] Hudson, N. W. , and R. C. Fraley . 2016. “Do People's Desires to Change Their Personality Traits Vary With Age? An Examination of Trait Change Goals Across Adulthood.” Social Psychological and Personality Science 7, no. 8: 847–856. 10.1177/1948550616657598.

[jopy13016-bib-0048] Hudson, N. W. , R. C. Fraley , W. J. Chopik , and D. A. Briley . 2020. “Change Goals Robustly Predict Trait Growth: A Mega‐Analysis of a Dozen Intensive Longitudinal Studies Examining Volitional Change.” Social Psychological and Personality Science 11, no. 6: 723–732. 10.1177/1948550619878423.

[jopy13016-bib-0049] Hudson, N. W. , and B. W. Roberts . 2014. “Goals to Change Personality Traits: Concurrent Links Between Personality Traits, Daily Behavior, and Goals to Change Oneself.” Journal of Research in Personality 53: 68–83. 10.1016/j.jrp.2014.08.008.

[jopy13016-bib-0050] Hudson, N. W. , and B. W. Roberts . 2016. “Social Investment in Work Reliably Predicts Change in Conscientiousness and Agreeableness: A Direct Replication and Extension of Hudson, Roberts, and Lodi‐Smith (2012).” Journal of Research in Personality 60: 12–23. 10.1016/j.jrp.2015.09.004. PMC3398702 22822278

[jopy13016-bib-0051] Jackson, J. J. , and A. J. Wright . 2024. “The Process and Mechanisms of Personality Change.” Nature Reviews Psychology 3, no. 5: 1–14. 10.1038/s44159-024-00295-z.

[jopy13016-bib-0120] Küchler, G. , K. S. A. Borgdorf , C. Aguilar‐Raab , and C. Wrzus . 2025. Self‐Reflections Across the Adult Lifespan: Associations with Personality Traits in a Binational Sample [Manuscript under revision]. Heidelberg University.

[jopy13016-bib-0052] Lakens, D. 2022. “Sample Size Justification.” Collabra: Psychology 8, no. 1: 33267. 10.1525/collabra.33267.

[jopy13016-bib-0053] Lamers, S. M. , G. J. Westerhof , V. Kovács , and E. T. Bohlmeijer . 2012. “Differential Relationships in the Association of the Big Five Personality Traits With Positive Mental Health and Psychopathology.” Journal of Research in Personality 46, no. 5: 517–524. 10.1016/j.jrp.2012.05.012.

[jopy13016-bib-0054] Leiner, D. J. 2021. “SoSci Survey (version 3.1.06).” https://www.soscisurvey.de.

[jopy13016-bib-0055] Malik, M. 1996. “Heart Rate Variability: Standards of Measurement, Physiological Interpretation, and Clinical Use: Task Force of the European Society of Cardiology and the North American Society for Pacing and Electrophysiology.” Annals of Noninvasive Electrocardiology 1, no. 2: 151–181. 10.1111/j.1542-474X.1996.tb00275.x.

[jopy13016-bib-0056] Mata, R. , A. K. Josef , G. R. Samanez‐Larkin , and R. Hertwig . 2011. “Age Differences in Risky Choice: A Meta‐Analysis.” Annals of the New York Academy of Sciences 1235, no. 1: 18–29. 10.1111/j.1749-6632.2011.06200.x. 22023565 PMC3332530

[jopy13016-bib-0057] Matsunaga, M. 2008. “Item Parceling in Structural Equation Modeling: A Primer.” Communication Methods and Measures 2, no. 4: 260–293. 10.1080/19312450802458935.

[jopy13016-bib-0058] McAdams, D. P. 2013. “The Psychological Self as Actor, Agent, and Author.” Perspectives on Psychological Science 8, no. 3: 272–295. 10.1177/1745691612464657. 26172971

[jopy13016-bib-0059] McComb, C. A. , E. J. Vanman , and S. J. Tobin . 2023. “A Meta‐Analysis of the Effects of Social Media Exposure to Upward Comparison Targets on Self‐Evaluations and Emotions.” Media Psychology 26, no. 5: 612–635. 10.1080/15213269.2023.2180647.

[jopy13016-bib-0115] McCrae, R. R. , P. T. Costa , M. P. de Lima , et al. 1999. “Age Differences in Personality Across the Adult Life Span: Parallels in Five Cultures.” Developmental Psychology 35, no. 2: 466–477. 10.1037/0012-1649.35.2.466. 10082017

[jopy13016-bib-0061] McLean, K. C. 2008. “Stories of the Young and the Old: Personal Continuity and Narrative Identity.” Developmental Psychology 44, no. 1: 254–264. 10.1037/0012-1649.44.1.254. 18194024

[jopy13016-bib-0062] McNiel, J. M. , and W. Fleeson . 2006. “The Causal Effects of Extraversion on Positive Affect and Neuroticism on Negative Affect: Manipulating State Extraversion and State Neuroticism in an Experimental Approach.” Journal of Research in Personality 40, no. 5: 529–550. 10.1016/j.jrp.2005.05.003.

[jopy13016-bib-0063] Meade, A. W. , and S. B. Craig . 2012. “Identifying Careless Responses in Survey Data.” Psychological Methods 17, no. 3: 437–455. 10.1037/a0028085. 22506584

[jopy13016-bib-0064] Mehlsen, M. , M. B. Mikkelsen , C. M. Andersen , and C. Ollars . 2019. “Does Aging and Disease Increase the Importance of Cognitive Strategies? Social and Temporal Comparisons in Healthy Younger and Older Adults and in Younger and Older Cancer Patients.” International Journal of Aging and Human Development 88, no. 1: 60–81. 10.1177/0091415017748366. 29278918

[jopy13016-bib-0065] Mendonça, S. E. , E. M. Dykhuis , and E. Jayawickreme . 2023. “Examining the Possibilities for Volitional Character Change in Compassion and Intellectual Humility Through a Three‐Month Online Intervention.” Journal of Research in Personality 104: 104373. 10.1016/j.jrp.2023.104373.

[jopy13016-bib-0066] Midgley, C. , S. Thai , P. Lockwood , C. Kovacheff , and E. Page‐Gould . 2021. “When Every Day Is a High School Reunion: Social Media Comparisons and Self‐Esteem.” Journal of Personality and Social Psychology 121, no. 2: 285–307. 10.1037/pspi0000336. 32790470

[jopy13016-bib-0067] Möller, J. , and H. W. Marsh . 2013. “Dimensional Comparison Theory.” Psychological Review 120, no. 3: 544–560. 10.1037/a0032459. 23544443

[jopy13016-bib-0068] Morina, N. 2021. “Comparisons Inform Me Who I Am: A General Comparative‐Processing Model of Self‐Perception.” Perspectives on Psychological Science 16, no. 6: 1281–1299. 10.1177/1745691620966788. 33615898 PMC8564255

[jopy13016-bib-0119] Movisens GmbH . n.d. “EcgMove4.” https://docs.movisens.com/Sensors/EcgMove4/#welcome.

[jopy13016-bib-0069] Movisens GmbH . 2019. DataAnalyzer Version 1.13.5 [Computer Software]. Movisens GmbH.

[jopy13016-bib-0070] Mund, M. , and F. J. Neyer . 2014. “Treating Personality‐Relationship Transactions With Respect: Narrow Facets, Advanced Models, and Extended Time Frames.” Journal of Personality and Social Psychology 107, no. 2: 352–368. 10.1037/a0036719. 25090133

[jopy13016-bib-0071] Muthén, L. K. , and B. O. Muthén . 1998–2017. Mplus User's Guide. 8th ed. Muthén & Muthén.

[jopy13016-bib-0072] Mutter, S. A. , J. M. Holder , C. A. Mashburn , and C. M. Luna . 2019. “Aging and the Role of Attention in Associative Learning.” Psychology and Aging 34, no. 2: 215–227. 10.1037/pag0000277. 30058825

[jopy13016-bib-0073] Ormel, J. , M. VonKorff , B. F. Jeronimus , and H. Riese . 2017. “Personality: Developmental Perspectives.” In Personality Development Across the Lifespan, edited by J. Specht , 117–137. Elsevier Academic Press.

[jopy13016-bib-0074] Pals, J. L. 2006. “Narrative Identity Processing of Difficult Life Experiences: Pathways of Personality Development and Positive Self‐Transformation in Adulthood.” Journal of Personality 74, no. 4: 1079–1110. 10.1111/j.1467-6494.2006.00403.x. 16787429

[jopy13016-bib-0075] Pasupathi, M. , and E. Mansour . 2006. “Adult Age Differences in Autobiographical Reasoning in Narratives.” Developmental Psychology 42, no. 5: 798–808. 10.1037/0012-1649.42.5.798. 16953687

[jopy13016-bib-0076] Pasupathi, M. , T. Weeks , and C. Rice . 2006. “Reflecting on Life: Remembering as a Major Process in Adult Development.” Journal of Language and Social Psychology 25, no. 3: 244–263. 10.1177/0261927X06289425.

[jopy13016-bib-0077] Pletzer, J. L. , I. Thielmann , and I. Zettler . 2024. “Who Is Healthier? A Meta‐Analysis of the Relations Between the HEXACO Personality Domains and Health Outcomes.” European Journal of Personality 38, no. 2: 342–364. 10.1177/08902070231174574.

[jopy13016-bib-0078] Pollock, E. R. , M. D. Young , D. R. Lubans , N. Eather , and P. J. Morgan . 2023. “Effects of a Father–Daughter Physical Activity Intervention Delivered by Trained Facilitators in the Community Setting on Girls' Social‐Emotional Well‐Being: A Randomized Controlled Trial.” Developmental Psychology 59, no. 10: 1852–1866. 10.1037/dev0001609. 37768618

[jopy13016-bib-0079] Post, K. 2004. “Die Achtsamkeitsbasierte Kognitive Therapie der Depression: Differenzielle Wirkmechanismen von Aufmerksamkeitsübungen und Verhaltensaktivierung.” Unpublished Diploma Thesis, University Münster.

[jopy13016-bib-0080] Quintus, M. , B. Egloff , and C. Wrzus . 2017. “Predictors of Volitional Personality Change in Younger and Older Adults: Response Surface Analyses Signify the Complementary Perspectives of the Self and Knowledgeable Others.” Journal of Research in Personality 70: 214–228. 10.1016/j.jrp.2017.08.001.

[jopy13016-bib-0081] Quintus, M. , B. Egloff , and C. Wrzus . 2021. “Daily Life Processes Predict Long‐Term Development in Explicit and Implicit Representations of Big Five Traits: Testing Predictions From the TESSERA (Triggering Situations, Expectancies, States and State Expressions, and ReActions) Framework.” Journal of Personality and Social Psychology 120, no. 4: 1049–1073. 10.1037/pspp0000361. 32955272

[jopy13016-bib-0082] Richetin, J. , G. Costantini , M. Perugini , and F. Schönbrodt . 2015. “Should We Stop Looking for a Better Scoring Algorithm for Handling Implicit Association Test Data? Test of the Role of Errors, Extreme Latencies Treatment, Scoring Formula, and Practice Trials on Reliability and Validity.” PLoS One 10, no. 6: e0129601. 26107176 10.1371/journal.pone.0129601 PMC4481268

[jopy13016-bib-0083] Riedl, R. , H. Kindermann , A. Auinger , and A. Javor . 2012. “Technostress From a Neurobiological Perspective: System Breakdown Increases the Stress Hormone Cortisol in Computer Users.” Business & Information Systems Engineering 4, no. 2: 61–69. 10.1007/s12599-012-0207-7.

[jopy13016-bib-0084] Roberts, B. W. , N. R. Kuncel , R. Shiner , A. Caspi , and L. R. Goldberg . 2007. “The Power of Personality: The Comparative Validity of Personality Traits, Socioeconomic Status, and Cognitive Ability for Predicting Important Life Outcomes.” Perspectives on Psychological Science 2, no. 4: 313–345. 10.1111/j.1745-6916.2007.00047.x. 26151971 PMC4499872

[jopy13016-bib-0114] Roberts, B. W. , J. Luo , D. A. Briley , P. I. Chow , R. Su , and P. L. Hill . 2017. “A Systematic Review of Personality Trait Change Through Intervention.” Psychological Bulletin 143, no. 2: 117–141. 10.1037/bul0000088. 28054797

[jopy13016-bib-0085] RStudio Team . 2021. RStudio: Integrated Development for R. RStudio. http://www.rstudio.com/.

[jopy13016-bib-0086] Rydell, R. J. , A. R. McConnell , and D. M. Mackie . 2008. “Consequences of Discrepant Explicit and Implicit Attitudes: Cognitive Dissonance and Increased Information Processing.” Journal of Experimental Social Psychology 44, no. 6: 1526–1532. 10.1016/j.jesp.2008.07.006.

[jopy13016-bib-0087] Sandstrom, G. M. , and E. J. Boothby . 2021. “Why Do People Avoid Talking to Strangers? A Mini Meta‐Analysis of Predicted Fears and Actual Experiences Talking to a Stranger.” Self and Identity 20, no. 1: 47–71. 10.1080/15298868.2020.1816568.

[jopy13016-bib-0088] Schmukle, S. C. , M. D. Back , and B. Egloff . 2008. “Validity of the Five‐Factor Model for the Implicit Self‐Concept of Personality.” European Journal of Psychological Assessment 24, no. 4: 263–272. 10.1027/1015-5759.24.4.263.

[jopy13016-bib-0089] Schröder‐Abé, M. , A. Rudolph , and A. Schütz . 2007. “High Implicit Self‐Esteem Is Not Necessarily Advantageous: Discrepancies Between Explicit and Implicit Self‐Esteem and Their Relationship With Anger Expression and Psychological Health.” European Journal of Personality: Published for the European Association of Personality Psychology 21, no. 3: 319–339. 10.1002/per.626.

[jopy13016-bib-0090] Schupp, J. , S. Herrmann , P. Jaensch , and F. R. Lang . 2008. “Erfassung Kognitiver Leistungspotentiale Erwachsener im Sozio‐Oekonomischen Panel (SOEP) [Assessment of Cognitive Performance Potentials of Adults in the Socio‐Economic Panel] (No. 32).” DIW Data Documentation.

[jopy13016-bib-0091] Seger, C. A. 1994. “Implicit Learning.” Psychological Bulletin 115, no. 2: 163–196. 10.1037/0033-2909.115.2.163. 8165269

[jopy13016-bib-0092] Smillie, L. D. , J. Wilt , R. Kabbani , C. Garratt , and W. Revelle . 2015. “Quality of Social Experience Explains the Relation Between Extraversion and Positive Affect.” Emotion 15, no. 3: 339–349. 10.1037/emo0000047. 25603131

[jopy13016-bib-0093] Sneed, J. R. , and S. K. Whitbourne . 2003. “Identity Processing and Self‐Consciousness in Middle and Later Adulthood.” Journals of Gerontology. Series B, Psychological Sciences and Social Sciences 58, no. 6: P313–P319. 10.1093/geronb/58.6.P313. 14614115

[jopy13016-bib-0094] Soto, C. J. , and O. P. John . 2017. “The Next Big Five Inventory (BFI‐2): Developing and Assessing a Hierarchical Model With 15 Facets to Enhance Bandwidth, Fidelity, and Predictive Power.” Journal of Personality and Social Psychology 113, no. 1: 117–143. 10.1037/pspp0000096. 27055049

[jopy13016-bib-0095] Speer, M. E. , and M. R. Delgado . 2017. “Reminiscing About Positive Memories Buffers Acute Stress Responses.” Nature Human Behaviour 1, no. 5: 0093. 10.1038/s41562-017-0093. PMC6719713 31482135

[jopy13016-bib-0096] Stieger, M. , C. Flückiger , D. Rüegger , T. Kowatsch , B. W. Roberts , and M. Allemand . 2021. “Changing Personality Traits With the Help of a Digital Personality Change Intervention.” Proceedings of the National Academy of Sciences of the United States of America 118, no. 8: e2017548118. 10.1073/pnas.2017548118. 33558417 PMC7923371

[jopy13016-bib-0097] Suls, J. , and R. Martin . 2005. “The Daily Life of the Garden‐Variety Neurotic: Reactivity, Stressors Exposure, Mood Spillover, and Maladaptive Coping.” Journal of Personality 73, no. 6: 1485–1510. 10.1111/j.1467-6494.2005.00356.x. 16274443

[jopy13016-bib-0098] Trapnell, P. D. , and J. D. Campbell . 1999. “Private Self‐Consciousness and the Five‐Factor Model of Personality: Distinguishing Rumination From Reflection.” Journal of Personality and Social Psychology 76, no. 2: 284–304. 10.1037/0022-3514.76.2.284. 10074710

[jopy13016-bib-0099] Wagg, E. , F. M. Blyth , R. G. Cumming , and S. Khalatbari‐Soltani . 2021. “Socioeconomic Position and Healthy Ageing: A Systematic Review of Cross‐Sectional and Longitudinal Studies.” Ageing Research Reviews 69: 101365. 10.1016/j.arr.2021.101365. 34004378

[jopy13016-bib-0100] Wagner, J. , N. Ram , J. Smith , and D. Gerstorf . 2016. “Personality Trait Development at the End of Life: Antecedents and Correlates of Mean‐Level Trajectories.” Journal of Personality and Social Psychology 111, no. 3: 411–429. 10.1037/pspp0000071. 26479363

[jopy13016-bib-0101] Wayment, H. A. , and S. E. Taylor . 1995. “Self‐Evaluation Processes: Motives, Information Use, and Self‐Esteem.” Journal of Personality 63, no. 4: 729–757. 10.1111/j.1467-6494.1995.tb00315.x. 8531044

[jopy13016-bib-0102] Weber, C. , M. Quintus , B. Egloff , G. Luong , M. Riediger , and C. Wrzus . 2020. “Same Old, Same Old? Age Differences in the Diversity of Daily Life.” Psychology and Aging 35, no. 3: 434–448. 10.1037/pag0000407. 31613136 PMC8021140

[jopy13016-bib-0103] Wilson, A. E. , and M. Ross . 2000. “The Frequency of Temporal‐Self and Social Comparisons in People's Personal Appraisals.” Journal of Personality and Social Psychology 78, no. 5: 928–942. 10.1037/0022-3514.78.5.928. 10821199

[jopy13016-bib-0104] Wilson, A. E. , and M. Ross . 2001. “From Chump to Champ: People's Appraisals of Their Earlier and Present Selves.” Journal of Personality and Social Psychology 80, no. 4: 572–584. 10.1037/0022-3514.80.4.572. 11316222

[jopy13016-bib-0105] Wolff, F. , F. Helm , F. Zimmermann , G. Nagy , and J. Möller . 2018. “On the Effects of Social, Temporal, and Dimensional Comparisons on Academic Self‐Concept.” Journal of Educational Psychology 110, no. 7: 1005–1025. 10.1037/edu0000248.

[jopy13016-bib-0118] Wrzus, C. , G. Küchler , K. S. A. Borgdorf , and C. Aguilar‐Raab . 2025. Less extraverted than others, but better looking than in the past… Domain‐specificity and temporal stability of social and temporal comparisons [Manuscript under revision]. Heidelberg University.

[jopy13016-bib-0106] Wrzus, C. , V. Müller , G. G. Wagner , U. Lindenberger , and M. Riediger . 2014. “Affect Dynamics Across the Lifespan: With Age, Heart Rate Reacts Less Strongly, but Recovers More Slowly From Unpleasant Emotional Situations.” Psychology and Aging 29, no. 3: 563–576. 10.1037/a0037451. 25244476

[jopy13016-bib-0107] Wrzus, C. , M. Quintus , and B. Egloff . 2023. “Age and Context Effects in Personality Development: A Multimethod Perspective.” Psychology and Aging 38, no. 1: 1–16. 10.1037/pag0000705. 36048045

[jopy13016-bib-0108] Wrzus, C. , and B. W. Roberts . 2017. “Processes of Personality Development in Adulthood: The TESSERA Framework.” Personality and Social Psychology Review 21, no. 3: 253–277. 10.1177/1088868316652279. 27260302

[jopy13016-bib-0109] Yentes, R. D. , and F. Wilhelm . 2018. “Careless: Procedures for Computing Indices of Careless Responding.” R Package Version 1[3].

[jopy13016-bib-0110] Van Zalk, M. H. W. , S. Nestler , K. Geukes , R. Hutteman , and M. D. Back . 2020. “The Codevelopment of Extraversion and Friendships: Bonding and Behavioral Interaction Mechanisms in Friendship Networks.” Journal of Personality and Social Psychology 118, no. 6: 1269–1290. 10.1037/pspp0000253. 31380681

[jopy13016-bib-0111] Zaw, S. , and M. Baldwin . 2023. “Knowing Who I Am Depends on Who I've Become: Linking Self‐Concept Clarity and Temporal Self‐Comparison.” Self and Identity 22, no. 6: 1281–1299. 10.1080/15298868.2023.2244722.

[jopy13016-bib-0112] Zell, E. , and M. D. Alicke . 2009. “Self‐Evaluative Effects of Temporal and Social Comparison.” Journal of Experimental Social Psychology 45, no. 1: 223–227. 10.1016/j.jesp.2008.09.007.

[jopy13016-bib-0113] Zell, E. , J. E. Strickhouser , C. Sedikides , and M. D. Alicke . 2020. “The Better‐Than‐Average Effect in Comparative Self‐Evaluation: A Comprehensive Review and Meta‐Analysis.” Psychological Bulletin 146, no. 2: 118–149. 10.1037/bul0000218. 31789535

