# Supplementary material for: Effects of Reflective Processes on Social–Emotional Trait Development in Adulthood: Insights From Two Multi‐Method Studies
Source: J Pers. 2025 Mar 6;94(1):60–80. doi: 10.1111/jopy.13016 (PMC12780306; doi:10.1111/jopy.13016)
Supplement: Supplementary file 1 — Appendix S1 [file JOPY-94-60-s001.docx]

**Supplementary material of "Effects of Reflective Processes on Social-Emotional Trait Development in Adulthood: Insights From Two Multi-Method Studies"**

[Study 1: Deviations From the Preregistration 1](#_Toc185415037)

[Study 1: Sociodemographic Information 3](#_Toc185415038)

[Study 1: Dropout-Analyses 5](#_Toc185415039)

[R Packages Used 6](#_Toc185415040)

[Study 1: Testing Measurement Invariance 7](#_Toc185415041)

[Study 1: Testing Cross-Country Differences 8](#_Toc185415042)

[Study 1: Additional Control Analyses With General Self-Reflections 9](#_Toc185415043)

[Study 2: Deviations From the Preregistration 10](#_Toc185415044)

[Study 2: Sociodemographic Information 11](#_Toc185415045)

[Study1: Dropout-Analyses 13](#_Toc185415046)

[Study 2: Latent Change Model 14](#_Toc185415047)

[Study 2: Testing Measurement Invariance 14](#_Toc185415048)

[Study 2: Results of Manipulation Checks 15](#_Toc185415049)

[Study 2: Robustness Checks 19](#_Toc185415050)

[References 19](#_Toc185415051)

##### Study 1: Deviations From the Preregistration

Table S1

*Study 1: Deviations From the Preregistered Hypothesis*

| **Original hypothesis** | **Hypothesis in the manuscript** | **Reason for changes** |
| --- | --- | --- |
| H1a: With more pronounced initial frequency of temporal comparisons, the explicit and implicit self-concepts of emotional stability and extraversion change more strongly over time. | H1a: With **a** more pronounced initial frequency of **past-temporal** comparisons, the explicit and implicit self-concepts of emotional stability and extraversion change more strongly over time (H1a). | Changed wording for grammatical correctness and consistency of the term past-temporal instead of temporal. |
| H1b: With more pronounced initial frequency of social comparisons, the explicit (but not implicit) self-concept of emotional stability and extraversion change more strongly over time. | H1b: With **a** more pronounced initial frequency of social comparisons, the explicit (but not implicit) **self-concepts** of **these traits** **change** more strongly over time (H1b) | Changed wording for grammatical correctness and redundancy. |
| H2: Effects of comparison standards on change in explicit self-concepts differ for emotional stability and extraversion: Changes in extraversion are more pronounced with a more pronounced initial frequency of social comparisons relative to past-temporal comparisons. We expect no differences between both comparison standards for changes in emotional stability. | H2: Changes in extraversion **would be** more pronounced with a more pronounced initial frequency of social comparisons relative to past-temporal comparisons. We **expected** no differences between both comparison standards for changes in emotional stability (H3). | Omitted some wording because of redundancy, changed wording for grammatical correctness |
| H3: The initial frequency of temporal comparisons moderates the correlated change between the explicit and implicit self-concepts of emotional stability and extraversion, respectively. With a more pronounced frequency of temporal comparisons, the correlated change of the explicit and implicit self-concepts of these traits is more strongly positive. |  | We did not investigate this hypothesis as it is beyond the scope of this paper. |
| H4: Changes in explicit and implicit self-concepts of emotional stability and extraversion are more pronounced among younger compared to older adults. | **H3**: Changes in explicit and implicit self-concepts of emotional stability and extraversion are more pronounced among younger individuals compared to older individuals. | Changed number of hypothesis.  Changed the terms of younger and older adults as they refer in literature to discrete age groups but age was assessed continuously in this study. |
| *Notes*. H = hypothesis. Bold text indicates that the wording has been changed without conceptual changes. | | |

##### Study 1: Sociodemographic Information

Table S2

*Study 1: Sociodemographic Information*

|  | USA | GE | Overall |
| --- | --- | --- | --- |
|  | % | % | % |
| Gender |  |  |  |
| Female | 56.8 | 50.9 | 52.4 |
| Male | 40.7 | 48.2 | 46.3 |
| Diverse (e.g., non-binary) | 2.5 | 0.4 | 1.0 |
| Age (in years) |  |  |  |
| 18‍–‍30 | 19.8 | 18.0 | 18.4 |
| 31‍–‍44 | 35.8 | 28.5 | 30.4 |
| 45‍–‍58 | 33.3 | 29.4 | 30.4 |
| 59‍–‍72 | 11.1 | 21.9 | 19.1 |
| 73‍–‍84 | 0.0 | 2.2 | 1.6 |
| Education |  |  |  |
| No degree | 0.0 | 0.0 | 0.0 |
| No college/university degree | 30.9 | 54.0 | 33.3 |
| College/university degree | 75.7 | 45.2 | 65.4 |
| Other | 2.5 | 0.9 | 1.3 |
| Occupation |  |  |  |
| Student or trainee | 4.9 | 9.6 | 8.4 |
| Full-time employee or public official | 30.9 | 31.6 | 31.4 |
| Part-time employee or public official | 13.6 | 12.7 | 12.9 |
| Self-employment | 32.1 | 23.2 | 25.6 |
| Marginal employment | 4.9 | 2.6 | 3.2 |
| Pensioner | 3.7 | 11.0 | 9.1 |
| Unemployed or seeking work | 7.4 | 7.5 | 7.4 |
| Other | 2.5 | 1.8 | 1.9 |
| Civil status |  |  |  |
| Married | 40.7 | 30.3 | 33.0 |
| Commited relationship | 12.3 | 23.2 | 20.4 |
| Divorced/separated, currently no relationship | 8.6 | 9.6 | 9.4 |
| Widowed, currently no relationship | 1.2 | 2.6 | 2.3 |
| Single, currently no relationship | 37.0 | 34.2 | 25.2 |
| Residence size (as number of inhabitants) |  |  |  |
| <2.000 | 12.3 | 5.3 | 7.1 |
| 2.000‍–‍5.000 | 8.6 | 10.1 | 9.7 |
| 5.001‍–‍20.000 | 12.3 | 16.7 | 15.5 |
| 20.001‍–‍50.000 | 12.3 | 16.2 | 15.2 |
| 50.001‍–‍100.000 | 6.2 | 10.5 | 9.4 |
| 100.001‍–‍500.000 | 12.3 | 18.0 | 16.5 |
| >500.000 | 29.6 | 23.2 | 24.9 |
| No information | 6.2 | 0.0 | 1.6 |
| Net income (in $ / €) |  |  |  |
| <1.000 | 23.5 | 23.2 | 23.3 |
| 1.000‍–‍2.000 | 22.2 | 26.8 | 25.6 |
| 2.000‍–‍3.000 | 9.9 | 27.6 | 23.0 |
| 3.000‍–‍5.000 | 21.0 | 7.5 | 11.0 |
| 5.000‍–‍7.500 | 4.9 | 1.3 | 2.3 |
| 7.500‍–‍10.000 | 2.5 | 0.0 | 0.6 |
| >10.000 | 4.9 | 0.0 | 1.3 |
| No information | 11.1 | 13.6 | 12.9 |
| Ethnicity^1^ |  |  |  |
| White/ Caucasian | 71.6 |  |  |
| Black/ African American | 12.3 |  |  |
| Latino/ Hispanic American | 3.7 |  |  |
| Asian American | 9.9 |  |  |
| Other | 2.5 |  |  |

*Note.* USA = United States, GE = Germany. ^1^Ethnicity was only assessed in the United States.

##### Study 1: Dropout-Analyses

| Table S3  *Dropout Analyses of Study 1* | | | | |
| --- | --- | --- | --- | --- |
| Variables *M(SD)* | Completers (*N* = 309) | Non-Completers  (*N* = 295) | *t(df)* or *Chi²* | *p* |
| Extraversion |  |  |  |  |
| Explicit | 2.96 (0.69) | 2.99 (0.72) | -0.52 (596.44) | .605 |
| Implicit | -.23 (0.51) | -0.23 (0.49) | 0.04 (583.28) | .969 |
| Emotional Stability |  |  |  |  |
| Explicit | 3.20 (0.79) | 3.05 (0.83) | 2.16 (597.25) | **.031** |
| Variables *M(SD)* | Completers (*N* = 309) | Non-Completers  (*N* = 295) | *t(df)* or *Chi²* | *p* |
| Implicit | 0.34 (0.36) | 0.26 (0.36) | 2.89 (579.35) | **.004** |
| Social Comparisons EX | 3.52 (1.58) | 3.81 (1.51) | -2.30 (601.99) | **.022** |
| Social Comparisons ES | 3.25 (1.50) | 3.31 (1.56) | -0.50 (597.68) | .616 |
| Past-temporal Comparisons EX | 3.45 (1.57) | 3.63 (1.51) | -1.41 (601.91) | .158 |
| Past-temporal Comparisons ES | 3.10 (1.55) | 3.39 (1.62) | -2.19 (597.41) | **.029** |
| Age | 45.01 (14.03) | 39.87 (14.43) | 4.44 (598.70) | **< .001** |
| Gender |  |  | 0.72 (1.00) | .397 |
| Female | 162 | 143 |  |  |
| Male | 144 | 148 |  |  |
| *Note*. EX =Extraversion, ES = Emotional Stability. Significant *p-*values (*p* < .05) are bolded. | | | | |

##### R Packages Used

| Table S4  *R Packages Employed for Data Preperation and Analyses* | |
| --- | --- |
| **Package** | **Reference/Source** |
| **dplyr** | Wickham, H., François, R., Henry, L., & Müller, K. (2020). dplyr: A Grammar of Data Manipulation (Version 1.0.5) [R package]. Retrieved from https://CRAN.R-project.org/package=dplyr |
| **tidyr** | Wickham, H. & Henry, L. (2020). tidyr: Tidy Messy Data (Version 1.1.3) [R package]. Retrieved from https://CRAN.R-project.org/package=tidyr |
| psych | Revelle, W. (2021). psych: Procedures for Psychological, Psychometric, and Personality Research (Version 2.1.6) [R package]. Retrieved from https://CRAN.R-project.org/package=psych |
| **plyr** | Wickham, H. (2011). plyr: Tools for Splitting, Applying and Combining Data (Version 1.8.6) [R package]. Retrieved from https://CRAN.R-project.org/package=plyr |
| **Package** | **Reference/Source** |
| **Hmisc** | Harrell Jr, F. E. & Dupont, C. (2022). Hmisc: Harrell Miscellaneous (Version 4.6.0) [R package]. Retrieved from https://CRAN.R-project.org/package=Hmisc |
| **MplusAutomation** | Hallquist, M. N. & Wiley, J. F. (2020). MplusAutomation: An R Package for Facilitating Large-Scale Latent Variable Analyses in Mplus (Version 0.7-7) [R package]. Retrieved from https://CRAN.R-project.org/package=MplusAutomation |
| **MBESS** | Kelley, K. (2021). MBESS: The MBESS R Package (Version 4.9.1) [R package]. Retrieved from https://CRAN.R-project.org/package=MBESS |
| **rstatix** | Kassambara, A. (2020). rstatix: Pipe-Friendly Framework for Basic Statistical Tests (Version 0.7.0) [R package]. Retrieved from https://CRAN.R-project.org/package=rstatix |
| **ggpubr** | Kassambara, A. (2020). ggpubr: 'ggplot2' Based Publication Ready Plots (Version 0.4.0) [R package]. Retrieved from https://CRAN.R-project.org/package=ggpubr |
| **car** | Fox, J. & Weisberg, S. (2019). car: Companion to Applied Regression (Version 3.0-10) [R package]. Retrieved from https://CRAN.R-project.org/package=car |
| **ggplot2** | Wickham, H. (2016). ggplot2: Elegant Graphics for Data Analysis (Version 3.3.5) [R package]. Retrieved from https://CRAN.R-project.org/package=ggplot2 |
| **gtable** | Wickham, H. (2016). gtable: Arrange grobs in tables (Version 0.3.0) [R package]. Retrieved from https://CRAN.R-project.org/package=gtable |
| **grid** | R Core Team. (2021). grid: The Grid Graphics Package (Version 4.1.1) [R package]. Retrieved from <https://www.R-project.org/> |
| **gridExtra** | Auguie, B. (2017). gridExtra: Miscellaneous Functions for "Grid" Graphics (Version 2.3) [R package]. Retrieved from https://CRAN.R-project.org/package=gridExtra |

#####

##### Study 1: Testing Measurement Invariance

We used the following guidelines of Chen (2007) for samples with *N* ≥300:

- To test configural invariance: Change of ≥.—.010 in CFI, supplemented by a change of ≥.015 in RMSEA or a change of ≥.030 in RMSEA compared to the less restrictive model would indicate noninvariance
- Metric invariance and strong invariance: change of ≥.—.010 in CFI, supplemented by a change of ≥.015 in RMSEA or a change of ≥.010 in SRMR compared to the less restrictive model would indicate noninvariance

| Table S5  *Study 1: Fit Indices for Models with Different Levels of Measurement Invariance* | | | | | |
| --- | --- | --- | --- | --- | --- |
| **Model** | χ*^2^* | CFI | TLI | RMSEA | SRMR |
| **ES explicit** |  |  |  |  |  |
| 1 | 9.930 | 0.997 | 0.991 | 0.056 | 0.010 |
| 2 | 12.502 | 0.997 | 0.993 | 0.050 | 0.017 |
| 3 | 13.354 | 0.997 | 0.996 | 0.040 | 0.017 |
| **EX explicit** |  |  |  |  |  |
| 1 | 8.185 | 0.998 | 0.993 | 0.045 | 0.010 |
| 2 | 8.472 | 0.999 | 0.998 | 0.026 | 0.011 |
| 3 | 8.585 | 1.000 | 1.000 | 0.000 | 0.011 |
| **ES implicit** |  |  |  |  |  |
| 1 | 0.329 | 1.000 | 1.000 | 0.000 | 0.004 |
| 2 | 1.453 | 1.000 | 1.000 | 0.000 | 0.014 |
| 3 | 2.091 | 1.000 | 0.999 | 0.012 | 0.018 |
| **EX implicit** |  |  |  |  |  |
| 1 | 4.287 | 0.994 | 0.966 | 0.103 | 0.006 |
| 2 | 4.652 | 0.995 | 0.986 | 0.066 | 0.007 |
| 3 | 1.953 | 1.000 | 1.000 | 0.000 | 0.009 |
| *Note.* CFI = Comparative fit index; TLI = Tucker-Lewis index; RMSEA = Root mean square error of approximation; SRMR = standardized root mean square residual. 1 = Unconstrained model/configural measurement invariance; 2 = Model with metric measurement invariance; 3 = Model with strong measurement invariance. | | | | | |

##### Study 1: Testing Cross-Country Differences

Table S6

*Study 1: Testing Cross-Country Differences*

| **Model** | | **Emotional stability** | | **Extraversion** | |
| --- | --- | --- | --- | --- | --- |
|  |  | Estimate [95% CI] | *p* | Estimate [95% CI] | *p* |
| **Explicit** | |  |  |  |  |
| M1 | Change T2–T1 | -0.009_a_ [-0.063, 0.046] | .758 | -0.042_a_ [-0.112, 0.028] | .237 |
|  | PTCOMP | 0.056 [0.011, ∞] | **.020** | 0.012 [-0.027, ∞] | .304 |
|  | Age | -0.033 [-∞, 0.008] | .090 | -0.024 [-∞, 0.040] | .270 |
|  | Country | -0.016_a_ [-0.072, 0.039] | .563 | 0.029_a_ [-0.041, 0.099] | .421 |
|  | PTCOMP by Country | -0.002_a_ [-0.054, 0.050] | .928 | 0.028_a_ [-0.046, 0.103] | .456 |
| M2 | Change T2–T1 | -0.010 _a_ [-0.066, 0.046] | .724 | -0.041_a_ [-0.110, 0.029] | .248 |
|  | SCOMP | 0.050 [0.006, ∞] | **.031** | -0.000 [-0.040, ∞] | .493 |
|  | Age | -0.032 [-∞, 0.009] | .100 | -0.025 [-∞, 0.040] | .267 |
|  | Country | -0.014_a_ [-0.071, 0.042] | .619 | 0.027_a_ [-0.043, 0.097] | .452 |
| **Model** | | **Emotional stability** | | **Extraversion** | |
|  |  | Estimate [95% CI] | *p* | Estimate [95% CI] | *p* |
|  | SCOMP by Country | -0.005_a_ [-0.056, 0.046] | .844 | 0.026_a_ [-0.047, 0.100] | .482 |
| M3 | Change T2–T1 | -0.004_a_ [-0.057, 0.049] | .880 | -0.041_a_ [-0.112, 0.029] | .251 |
|  | Age | -0.002 [-∞, 0.047] | .472 | -0.025 [ -∞, 0.038] | .254 |
|  | Country | -0.025_a_ [-0.079, 0.028] | .353 | 0.027_a_ [-0.044, 0.098] | .454 |
|  | Age by Country | -0.032_a_ [-0.020, 0.084] | .231 | 0.028_a_ [-0.047, 0.103] | .464 |
| **Implicit** | | |  |  |  |
| M1 | Change T2–T1 | 0.001_a_ [-0.050, 0.053] | .962 | 0.003_a_ [-0.059, 0.065] | .923 |
|  | PTCOMP | -0.006 _a_ [-0.048, 0.036] | .767 | -0.009_a_ [-0.058, 0.041] | .736 |
|  | Age | 0.019 [-∞, 0.054] | .183 | -0.017 [-∞, 0.031] | .278 |
|  | Country | -0.030_a_ [-0.082, 0.021] | .252 | -0.024_a_ [-0.085, 0.036] | .433 |
|  | PTCOMP by Country | 0.010_a_ [-0.032, 0.052] | .634 | 0.012_a_ [-0.036, 0.061] | .620 |
| M2 | Change T2–T1 | 0.001_a_ [-0.050, 0.053] | .965 | 0.003_a_ [-0.059, 0.065] | .922 |
|  | SCOMP | -0.005_a_ [-0.048, 0.038] | .826 | -0.009_a_ [-0.059, 0.040] | .718 |
|  | Age | 0.018 [-∞, 0.053] | .193 | -0.018 [-∞, 0.031] | .276 |
|  | Country | -0.030_a_ [-0.082, 0.021] | .250 | -0.024_a_ [-0.086, 0.037] | .436 |
|  | SCOMP by Country | -0.001_a_ [-0.044, 0.042] | .961 | 0.013_a_ [-0.034, 0.060] | .584 |
| M3 | Change T2–T1 | -0.004_a_ [-0.057, 0.049] | .880 | -0.015_a_ [-0.075, 0.045] | .632 |
|  | Age | -0.002 [-∞, 0.047] | .472 | -0.067 [-∞, -0.015] | **.017** |
|  | Country | -0.025_a_ [-0.079, 0.028] | .353 | -0.010_a_ [-0.070, 0.051] | .749 |
|  | Age by Country | 0.032_a_ [-0.020, 0.084] | .231 | 0.077_a_ [0.025, 0.129] | **.004** |
| *Note*. *N* = 309. PTCOMP = Past-temporal comparison, SCOMP = Social comparison, M1 = Model 1, M2 = Model 2, M3 = Model 3. _a_undirected hypothesis. Significant *p-*values (*p* < .05) are bolded. | | | | | |

##### Study 1: Additional Control Analyses With General Self-Reflections

| Table S7  *Study 1: Control Analyses with General Self-Reflections as Covariates* | | | | | |
| --- | --- | --- | --- | --- | --- |
| **Model** | | **Emotional stability** | | **Extraversion** | |
|  |  | Estimate [95% CI] | *p* | Estimate [95% CI] | *p* |
| **Explicit** | |  |  |  |  |
| M1 | Change T2–T1 | -0.001_a_ [-0.058, 0.056] | .970 | -0.039_a_ [-0.105, 0.027] | .245 |
|  | PTCOMP | 0.061 [0.021, ∞] | **.006** | 0.007 [-0.035, ∞] | .391 |
|  | Age | -0.044 [-∞, -0.001] | **.045** | -0.002 [-∞, 0.039] | .461 |
|  | Explorative SR | 0.016_a_ [-0.029, 0.061] | .478 | -0.004_a_ [-0.045, 0.038] | .863 |
|  | Ruminative SR | -0.060_a_ [-0.130, 0.009] | .089 | 0.024_a_ [-0.022, 0.069] | .308 |
| M2 | Change T2–T1 | -0.018_a_ [-0.073, 0.038] | .535 | -0.039_a_ [-0.105, 0.027] | .248 |
|  | SCOMP | 0.058 [0.018, ∞] | **.009** | -0.011 [-0.054, ∞] | .340 |
|  | Age | -0.042 [-∞, 0.000] | .050 | -0.004 [-∞, 0.038] | .439 |
|  | Explorative SR | 0.020_a_ [-0.023, 0.064] | .355 | 0.000_a_ [-0.041, 0.042] | .990 |
|  | Ruminative SR | -0.058_a_ [-0.127, 0.011] | .099 | 0.032_a_ [-0.015, 0.078] | .180 |
| *Note*. *N* = 309. PTCOMP = Past-temporal comparison, SCOMP = Social comparison, M1 = Model 1, M2 = Model 2, M3 = Model 3. SR = Self-reflection. _a_undirected hypothesis. Significant *p-*values (*p* < .05) are bolded. | | | | | |

##### Study 2: Deviations From the Preregistration

Table S8

*Study 2: Deviations from the Preregistered Hypotheses*

| **Original hypothesis** | **Hypothesis in the manuscript** | **Reason for changes** |
| --- | --- | --- |
| H1. Personality changes differ when based on past-temporal vs. social comparisons. |  | We omitted this hypothesis due to redundancy with H1a-b |
| H2a. Personality changes are more pronounced among younger adults compared to older adults. | **H3**a: Personality changes are more pronounced among younger adults compared to older adults (H3a). | We changed the number of the hypotheses to adapt them to the order of appearance in the manuscript. |
| H2b. Personality changes among younger adults are more pronounced when based on social comparisons compared to past-temporal comparisons, whereas personality changes among older adults are more pronounced based on past-temporal comparisons compared to social comparisons. | **H3b**: personality changes among younger adults are more pronounced when based on social comparisons compared to past-temporal comparisons, whereas personality changes among older adults are more pronounced based on past-temporal comparisons compared to social comparisons (H3b). |  |
| H3a. Explicit trait self-concepts change more compared to implicit trait self-concepts. H3b. Effects of comparison standards on personality change differ for explicit and implicit trait self-concepts: Changes in explicit trait self-concepts differ when based on past-temporal vs. social comparison, whereas changes in implicit self-concepts are not affected by the comparison standards. | **H2a**: Explicit trait self-concepts change more compared to implicit trait self-concepts **H2b**: Further, we expected that changes in implicit self-concepts are not affected by the comparison standards. | We changed the number of the hypothesis to adapt it to the order of appearance in the manuscript. We omitted text due to redundancy, the meaning remains the same |
| H4a. Changes in extraversion are more pronounced compared to changes in emotional stability. | **H1a**. Changes in extraversion are more pronounced compared to changes in emotional stability. | We changed the number of the hypothesis to adapt it to the order of appearance in the manuscript. |
| H4b. Effects of comparison standards on change in explicit personality differ for extraversion and emotional stability: Changes in extraversion are more pronounced after social comparisons relative to past-temporal comparisons. No difference is expected for emotional stability. | **H1b:** Changes in extraversion are more pronounced after social comparisons relative to past-temporal comparisons (…) no difference **was** expected for emotional stability. | We changed the number of the hypothesis and omitted text due to redundancy, the meaning remains the same. |
| *Notes*. H = Hypothesis. Bold text indicates that the number has been changed without conceptual changes. | | |

##### Study 2: Sociodemographic Information

Table S9

*Study 2: Sociodemographic Information*

|  | YA | OA | Overall |
| --- | --- | --- | --- |
|  | *M* (*SD*) | *M* (*SD*) | *M* (*SD*) |
| Age | 24.60 (3.90) | 71.00 (6.00) | 47.30 (23.80) |
|  | % | % | % |
| Gender |  |  |  |
| Female | 75.4 | 63.7 | 69.7 |
| Male | 22.0 | 36.3 | 29.0 |
| Diverse (e.g., non-binary) | 2.5 | 0.0 | 1.3 |
| Education |  |  |  |
| No degree | 0.0 | 0.9 | 0.4 |
| No college/university degree | 55.1 | 42.5 | 48.9 |
| College/university degree | 39.8 | 46.0 | 42.9 |
| Other | 5.1 | 10.6 | 7.8 |
| Occupation |  |  |  |
| Student or trainee | 83.9 | 0.9 | 43.3 |
| Full-time employee or public official | 3.4 | 2.7 | 3.0 |
| Part-time employee or public official | 5.1 | 8.0 | 6.5 |
| Self-employment | 0.0 | 4.4 | 2.2 |
| Marginal employment | 0.0 | 0.0 | 0.0 |
| Pensioner | 0.0 | 78.8 | 38.5 |
| Unemployed or seeking work | 1.7 | 0.9 | 1.3 |
| Other | 5.9 | 4.4 | 5.2 |
| Civil status |  |  |  |
| Married | 5.9 | 43.4 | 24.2 |
| Commited relationship | 40.7 | 12.4 | 26.8 |
| Divorced/separated, currently no relationship | 0.8 | 22.1 | 11.3 |
| Widowed, currently no relationship | 0.0 | 11.5 | 5.6 |
| Single, currently no relationship | 52.5 | 10.6 | 32.0 |
| Residence size (as number of inhabitants) |  |  |  |
| <2.000 | 0.0 | 3.5 | 1.7 |
| 2.000‍–‍5.000 | 0.8 | 5.3 | 3.0 |
| 5.001‍–‍20.000 | 16.9 | 30.1 | 23.4 |
| 20.001‍–‍50.000 | 6.8 | 15.0 | 10.8 |
| 50.001‍–‍100.000 | 0.8 | 0.9 | 0.9 |
| 100.001‍–‍500.000 | 71.2 | 45.1 | 58.4 |
| >500.000 | 0.0 | 0.0 | 0.0 |
| No information | 3.4 | 0.0 | 1.7 |
| Net income (in $ / €) |  |  |  |
| <1.000 | 56.8 | 8.8 | 33.3 |
| 1.000‍–‍2.000 | 18.6 | 26.5 | 22.5 |
| 2.000‍–‍3.000 | 2.5 | 30.1 | 16.0 |
| 3.000‍–‍5.000 | 2.5 | 25.7 | 13.9 |
| 5.000‍–‍7.500 | 0.0 | 2.7 | 1.3 |
| 7.500‍–‍10.000 | 0.0 | 0.9 | 0.4 |
| >10.000 | 0.0 | 0.0 | 0.0 |
| No information | 19.5 | 5.3 | 12.6 |

Note. YA = Younger adults, OA = Older adults.

##### Study1: Dropout-Analyses

| Table S10  *Study 2: Dropout Analyses* | | | | |
| --- | --- | --- | --- | --- |
| Variables *M(SD)* | Completers (*N* = 231) | Non-Completers  (*N* = 39) | *t(df)* or *Chi²* | *p* |
| Extraversion |  |  |  |  |
| Explicit | 3.34 (0.64) | 3.43 (0.67) | -0.80 (50.52) | .430 |
| Implicit | 0.01 (0.61) | 0.03 (0.55) | -0.30 (55.59) | .766 |
| Emotional Stability |  |  |  |  |
| Explicit | 3.29 (0.66) | 3.26 (0.64) | 0.22 (52.47) | .826 |
| Implicit | 0.39 (0.36) | 0.22 (0.29) | 3.06 (53.85) | **.003** |
| Age | 47.31 (23.76) | 43.08 (24.68) | 1.01 (52.30) | .318 |
| Gender (*n*) |  |  | 1.85 (1) | .174 |
| Female | 161 | 33 |  |  |
| Male | 67 | 7 |  |  |
| *Note*. EX =Extraversion, ES = Emotional Stability. Significant *p-*values (*p* < .05) are bolded. | | | | |

#####

##### Study 2: Latent Change Model

| 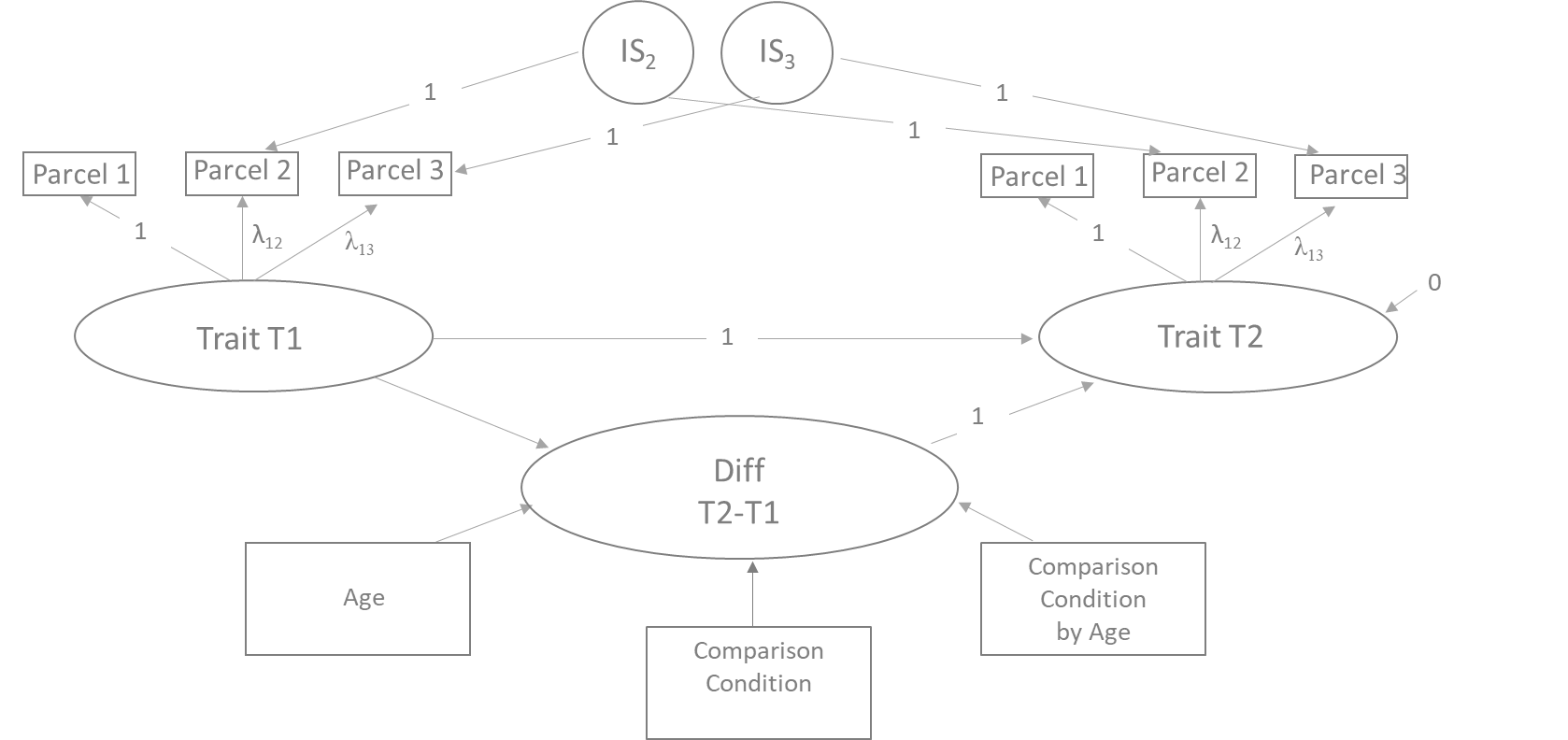 |
| --- |
| *Figure S1****.*** Latent change model of the explicit self-concepts. Latent traits were estimated with three indicators (parcels) for each at T1 and T2. Measurement invariance was achieved by constraining intercepts (not displayed) and factor loadings to be equal for each measurement. Repeated method effects were accounted for by indicator-specific method factors (IS2, IS3). The latent variable Diff T2–T1 reflects the amount of latent change in traits from T1 to T2. Latent trait change was predicted by age, comparison conditions, and their interaction. |

##### Study 2: Testing Measurement Invariance

We used the following guidelines of Chen (2007) for samples with *N* ≤ 300:

- To test configural invariance: Change of ≤—.005 in CFI, supplemented by a change of ≥.010 in RMSEA or a change of ≥.025 in RMSEA compared to the less restrictive model would indicate noninvariance
- Metric invariance and strong invariance: change of ≥ —.005 in CFI, supplemented by a change of ≥.015 in RMSEA or a change of ≥.010 in SRMR compared to the less restrictive model would indicate noninvariance

| Table S11  *Study 2: Fit Indices for Models with Different Levels of Measurement Invariance* | | | | | |
| --- | --- | --- | --- | --- | --- |
| **Model** | χ*^2^* | CFI | TLI | RMSEA | SRMR |
| **ES explicit** |  |  |  |  |  |
| 1 | 5.504 | 1.000 | 0.999 | 0.021 | 0.014 |
| 2 | 6.021 | 1.000 | 1.002 | 0.000 | 0.017 |
| 3 | 10.937 | 0.998 | 0.997 | 0.031 | 0.016 |
| **EX explicit** |  |  |  |  |  |
| 1 | 8.439 | 0.997 | 0.991 | 0.055 | 0.013 |
| 2 | 8.609 | 0.999 | 0.997 | 0.032 | 0.014 |
| 3 | 10.348 | 0.999 | 0.998 | 0.025 | 0.016 |
| **ES implicit** |  |  |  |  |  |
| 1 | 1.164 | 0.999 | 0.996 | 0.027 | 0.008 |
| 2 | 1.489 | 1.000 | 1.000 | 0.000 | 0.009 |
| 3 | 0.317 | 1.000 | 1.000 | 0.000 | 0.004 |
| **EX implicit** |  |  |  |  |  |
| 1 | 0.747 | 1.000 | 1.000 | 0.000 | 0.003 |
| 2 | 0.836 | 1.000 | 1.000 | 0.000 | 0.003 |
| 3 | 0.320 | 1.000 | 1.000 | 0.000 | 0.004 |
| *Note.* CFI = Comparative fit index; TLI = Tucker-Lewis index; RMSEA = Root mean square error of approximation; SRMR = Standardized root mean square residual. 1 = Unconstrained model/configural measurement invariance; 2 = Model with metric measurement invariance; 3 = Model with strong measurement invariance. | | | | | |

##### Study 2: Results of Manipulation Checks

| Table S12  *Study 2: Means and Standard Deviations and Results of t-Tests for Manipulation Check Variables* | | | | | |
| --- | --- | --- | --- | --- | --- |
| Variables *M(SD)* | PTCOMP | SCOMP | Younger adults | Older adults | Overall |
| **Affect** |  |  |  |  |  |
| Baseline | 5.30_a_ (1.27) | 5.30 _a_ (1.19) | 5.03_a_ (1.07) | 5.74_b_ (1.28) | 5.36 (1.23) |
| After Stressor | 5.18_a_ (1.35) | 5.03 _a_ (1.30) | 4.74_a_ (1.15) | 5.48_b_ (1.39) | 5.09 (1.33) |
| After Social Interaction | 5.71_a_ (1.20) | 5.72 _a_ (1.05) | 5.40_a_ (0.99) | 6.05_b_ (1.15) | 5.71 (1.11) |
| **Heart Rate** |  |  |  |  |  |
| Baseline | 79.09_a_ (12.99) | 77.86_a_ (14.19) | 80.07_a_ (13.66) | 76.58_a_ (13.41) | 78.43 (13.62) |
| After Stressor | 83.09_a_ (12.69) | 85.54_a_ (16.86) | 87.91_a_ (14.87) | 80.38_b_ (14.37) | 84.40 (15.08) |
| After Social Interaction | 79.28_a_ (10.90) | 79.56_a_ (13.64) | 81.46_a_ (12.54) | 77.08_b_ (11.84) | 79.43 (12.38) |
| **Rmssd** |  |  |  |  |  |
| Baseline | 32.95_a_ (20.79) | 34.80_a_ (22.96) | 41.01_a_ (20.19) | 23.87_b_ (20.14) | 33.85 (21.82) |
| After Stressor | 27.21_a_ (15.69) | 30.58_a_ (18.74) | 34.45_a_ (16.53) | 21.71_b_ (15.86) | 28.97 (17.38) |
| After Social Interaction | 29.26_a_ (16.46) | 32.51_a_ (18.62) | 39.34_a_ (16.99) | 19.88_b_ (11.17) | 30.92 (17.61) |
| **Trait-relevant states** |  |  |  |  |  |
| ES self-report | 5.75 _a_ (1.31) | 5.52 _a_ (1.19) | 5.19 _a_ (1.23) | 6.10_b_ (1.09) | 5.63 (1.25) |
| ES other-report | 6.02 _a_ (1.10) | 5.71_b_ (1.21) | 5.61_a_ (1.22) | 6.12_b_ (1.05) | 5.86 (1.15) |
| EX self-report | 5.88 _a_ (1.28) | 5.85 _a_ (1.19) | 5.45 _a_ (1.34) | 6.32 _b_ (0.91) | 5.87 (1.23) |
| EX other-report | 5.97 _a_ (1.10) | 5.89 _a_ (1.26) | 5.69 _a_ (1.33) | 6.19 _b_ (0.95) | 5.94 (1.16) |

*Note*. ES = Emotional stability, EX = Extraversion, SCOMP = Social comparison, PTCOMP = Past-temporal Comparison. Means with different subscripts differ significantly between comparison condition/age group, with *p* < .05.

| Table S13  *Study 2: Results of Mixed Effects ANOVAs Predicting the Change of Manipulation Check Variables by Age Group, Time and Their Interaction* | | | | | | | |
| --- | --- | --- | --- | --- | --- | --- | --- |
| **Predictor** | ***df_Num_*** | ***df_Den_*** | ***SS_Num_*** | ***SS_Den_*** | ***F*** | ***p*** | **η²_g_** |
| **Affect** |  |  |  |  |  |  |  |
| *Baseline vs. after stress induction* | | | | | | | |
| Intercept | 1 | 226 | 12526.085 | 602.624 | 4697.613 | **<.001** | .948 |
| Age | 1 | 226 | 59.085 | 602.624 | 22.158 | **<.001** | .080 |
| Time | 1 | 226 | 8.104 | 80.871 | 22.648 | **<.001** | .012 |
| Time by Age | 1 | 226 | 0.052 | 80.871 | 0.145 | .704 | .00007 |
| *After stress induction vs. after stress reduction* | | | | | | | |
| Intercept | 1 | 227 | 13401.569 | 531.914 | 5719.261 | **<.001** | .955 |
| Age | 1 | 227 | 53.951 | 531.914 | 23.024 | **<.001** | .079 |
| Time | 1 | 227 | 44.783 | 96.914 | 104.894 | **<.001** | .066 |
| Time by Age | 1 | 227 | 0.111 | 96.914 | 0.259 | .611 | .0002 |
| **Heart rate** |  |  |  |  |  |  |  |
| *Baseline vs. after stress induction* | | | | | | | |
| Intercept | 1 | 180 | 2375340.622 | 60726.663 | 7040.751 | **<.001** | .972 |
| Age | 1 | 180 | 2745.864 | 60726.663 | 8.139 | **.005** | .039 |
| Time | 1 | 180 | 3348.166 | 7285.051 | 82.727 | **<.001** | .047 |
| Time by Age | 1 | 180 | 128.380 | 7285.051 | 3.172 | .077 | .002 |
| *After stress induction vs. after stress reduction* | | | | | | | |
| Intercept | 1 | 172 | 2284343.070 | 52613.78 | 7467.758 | **<.001** | .976 |
| Age | 1 | 172 | 2624.621 | 52613.78 | 8.580 | **.004** | .044 |
| Time | 1 | 172 | 1460.241 | 3985.82 | 63.014 | **<.001** | .025 |
| Time by Age | 1 | 172 | 127.390 | 3985.82 | 5.497 | **.020** | .002 |
| **RMSSD** |  |  |  |  |  |  |  |
| *Baseline vs. after stress induction* | | | | | | | |
| Intercept | 1 | 131 | 227071.612 | 78112.427 | 380.815 | **<.001** | .725 |
| Age | 1 | 131 | 16973.149 | 78112.427 | 28.465 | **<.001** | .165 |
| Time | 1 | 131 | 1715.966 | 8014.599 | 28.048 | **<.001** | .020 |
| Time by Age | 1 | 131 | 83.741 | 8014.599 | 1.369 | .244 | .001 |
| *After stress induction vs. after stress reduction* | | | | | | | |
| Intercept | 1 | 132 | 206790.573 | 48693.132 | 560.579 | **<.001** | .790 |
| Age | 1 | 132 | 18159.879 | 48693.132 | 49.229 | **<.001** | .249 |
| Time | 1 | 132 | 549.789 | 6123.839 | 11.851 | **<.001** | .010 |
| Time by Age | 1 | 132 | 281.830 | 6123.839 | 6.075 | **.015** | .005 |
| *Note*. *df_Num_ = Degrees of freedom numerator, df_Den_ = Degrees of freedom denominator, SS_Num_= Sum of squares numerator, SS_Den_ = Sum of squares denominator.* η²_g_= Generalized eta-squared. Italicized text represents the analyzed experimental phases. Significant *p-*values (*p*< .05) are bolded. | | | | | | | |

| 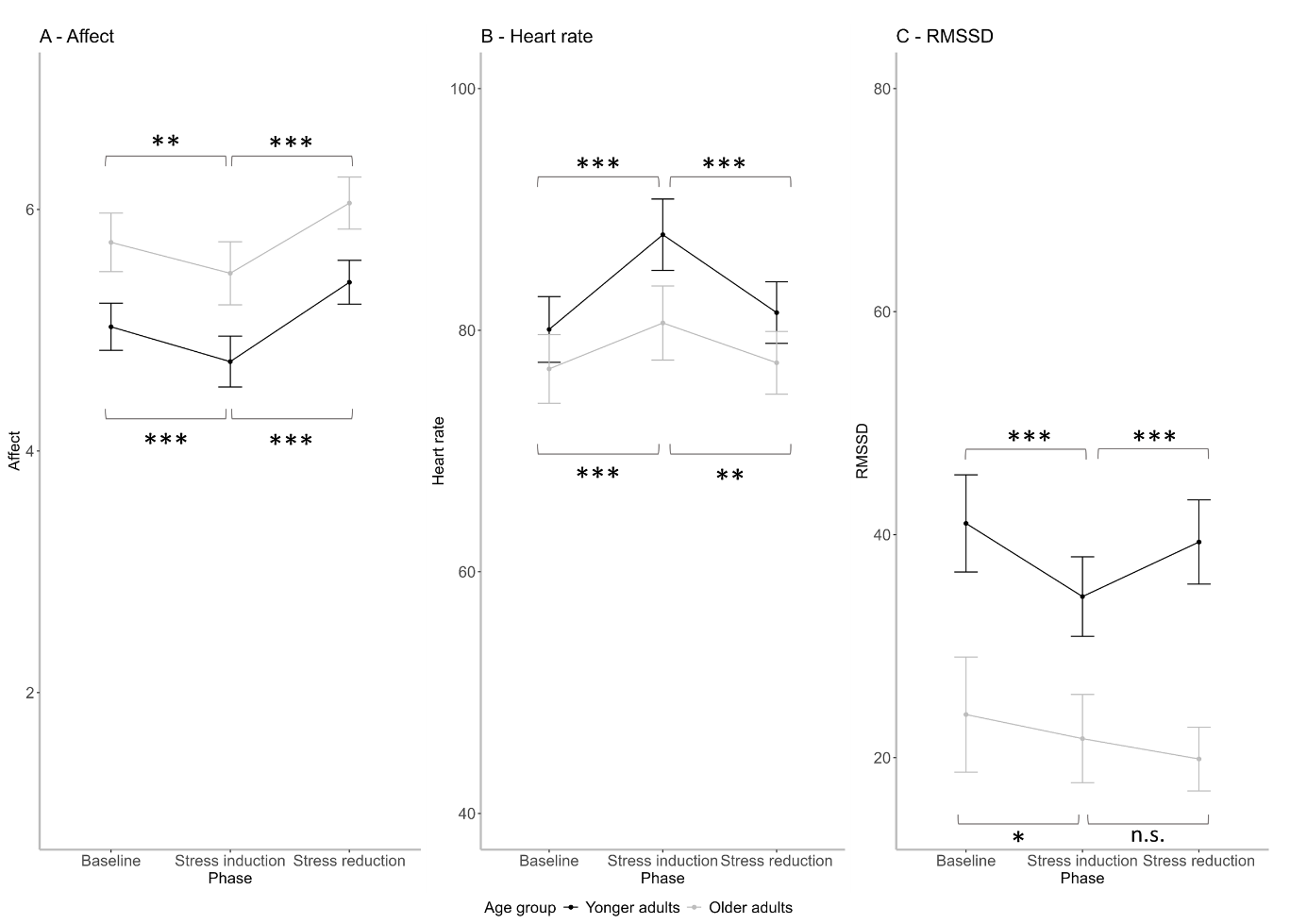 |
| --- |
| *Figure S2.* Change of affect (A), heart rate (B), and RMSSD (C) across the phases of the experiment, separately for younger and older adults. Error bars represent confidence intervals of the mean. Affect ratings represent the mean and were assessed before the baseline and after the stress induction and reduction. Parameters of cardiac activity were assessed continuously; data points represent the mean of each phase. Brackets and stars display Bonferroni-corrected results of pairwise comparisons within each age group.  *** *p* < .001, ***p* <. 01, **p* < .05, n.s. = not significant. |

##### Study 2: Robustness Checks

| Table S14  *Study 2: Robustness Checks of Differences of Effects Between Sessions with Participants vs. Confederate* | | | | |
| --- | --- | --- | --- | --- |
| **Model** | **Emotional stability** | | **Extraversion** | |
|  | Estimate [95% CI] | *p* | Estimate [95% CI] | *p* |
| **Explicit** |  |  |  |  |
| Change T2–T1 | 0.056 [0.017, ∞] | **.010** | 0.046 [0.005, ∞] | **.031** |
| COMP | 0.020_a_ [-0.023, 0.064] | .356 | -0.017 [-∞, 0.022] | .237 |
| Age | -0.007 [-∞, 0.040] | .403 | 0.025 [-∞, 0.074] | .196 |
| CONF | 0.00_a_ [-0.039, 0.052] | .775 | 0.004_a_ [-0.043, 0.051] | .874 |
| COMP by Age | -0.009 [-0.055, ∞] | .368 | 0.024 [-0.023, ∞] | .201 |
| CONF by Age | -0.034_a_ [-0.127,0.059] | .475 | -0.038_a_ [-0.135, 0.059] | .445 |
| COMP by Age by CONF | -0.021_a_ [-0.113, 0.070] | .647 | -0.030_a_ [-0.124, 0.064] | .534 |
| **Implicit** |  |  |  |  |
| Change T2–T1 | -0.046 [-0.089, ∞] | **.036** | 0.027 [-0.038, ∞] | .248 |
| COMP | -0.006_a_ [-0.043, 0.031] | .765 | 0.012_a_ [-0.047, 0.071] | .696 |
| AGE | 0.001 [-∞, 0.048] | .488 | 0.002 [-∞, 0.062] | .478 |
| CONF | -0.003_a_ [-0.043, 0.038] | .898 | 0.023 [-0.045, 0.090] | .511 |
| COMP by Age | 0.007_a_ [-0.039, 0.052] | .775 | -0.030_a_ [-0.095, 0.035] | .366 |
| CONF by Age | -0.072_a_ [-0.151, 0.006] | .070 | 0.037_a_ [-0.100, 0.175] | .594 |
| COMP by Age by COMP | -0.036_a_ [-0.114, 0.042] | .366 | 0.046_a_ [-0.080, 0.172] | .472 |
| *Note*. COMP = Comparison condition. Experimental conditions were coded as -1 = Social comparison, 1 = Past-temporal comparison. Age was coded as -1 = Younger adults and 1 = Older adults. CONF = Confederate was coded as -1 = Participant and 1 = Confederate present. Significant *p-*values (*p* < .05) are bolded. | | | | |

##### References

Chen, F. F. (2007). Sensitivity of Goodness of Fit Indexes to Lack of Measurement

Invariance. *Structural Equation Modeling: A Multidisciplinary Journal*, *14*(3), 464–504. https://doi.org/10.1080/10705510701301834
